# E3 ligases MAC3A and MAC3B ubiquitinate UBIQUITIN-SPECIFIC PROTEASE14 to regulate organ size in Arabidopsis

**DOI:** 10.1093/plphys/kiad559

**Published:** 2023-10-18

**Authors:** Xiaopeng Guo, Xin Zhang, Shan Jiang, Xin Qiao, Bolun Meng, Xiaohang Wang, Yanan Wang, Kaihuan Yang, Yilan Zhang, Na Li, Tianyan Chen, Yiyang Kang, Mengyi Yao, Xuan Zhang, Xinru Wang, Erling Zhang, Junhua Li, Dawei Yan, Zhubing Hu, José Ramón Botella, Chun-Peng Song, Yunhai Li, Siyi Guo

**Affiliations:** State Key Laboratory of Crop Stress Adaptation and Improvement, College of Life Sciences, Academy for Advanced Interdisciplinary Studies, Henan University, Kaifeng 475004, China; Sanya Institute, Henan University, Sanya 572025, China; State Key Laboratory of Crop Stress Adaptation and Improvement, College of Life Sciences, Academy for Advanced Interdisciplinary Studies, Henan University, Kaifeng 475004, China; Sanya Institute, Henan University, Sanya 572025, China; State Key Laboratory of Plant Cell and Chromosome Engineering, CAS Centre for Excellence in Molecular Plant Biology, Institute of Genetics and Developmental Biology, Chinese Academy of Sciences, Beijing 100101, China; State Key Laboratory of Crop Stress Adaptation and Improvement, College of Life Sciences, Academy for Advanced Interdisciplinary Studies, Henan University, Kaifeng 475004, China; State Key Laboratory of Crop Stress Adaptation and Improvement, College of Life Sciences, Academy for Advanced Interdisciplinary Studies, Henan University, Kaifeng 475004, China; State Key Laboratory of Crop Stress Adaptation and Improvement, College of Life Sciences, Academy for Advanced Interdisciplinary Studies, Henan University, Kaifeng 475004, China; State Key Laboratory of Crop Stress Adaptation and Improvement, College of Life Sciences, Academy for Advanced Interdisciplinary Studies, Henan University, Kaifeng 475004, China; State Key Laboratory of Crop Stress Adaptation and Improvement, College of Life Sciences, Academy for Advanced Interdisciplinary Studies, Henan University, Kaifeng 475004, China; State Key Laboratory of Crop Stress Adaptation and Improvement, College of Life Sciences, Academy for Advanced Interdisciplinary Studies, Henan University, Kaifeng 475004, China; State Key Laboratory of Plant Cell and Chromosome Engineering, CAS Centre for Excellence in Molecular Plant Biology, Institute of Genetics and Developmental Biology, Chinese Academy of Sciences, Beijing 100101, China; State Key Laboratory of North China Crop Improvement and Regulation, College of Horticulture, Hebei Agricultural University, Baoding 071000, China; State Key Laboratory of Plant Cell and Chromosome Engineering, CAS Centre for Excellence in Molecular Plant Biology, Institute of Genetics and Developmental Biology, Chinese Academy of Sciences, Beijing 100101, China; College of Life Sciences, Yunnan University, Kunming 650500, China; State Key Laboratory of Crop Stress Adaptation and Improvement, College of Life Sciences, Academy for Advanced Interdisciplinary Studies, Henan University, Kaifeng 475004, China; State Key Laboratory of Crop Stress Adaptation and Improvement, College of Life Sciences, Academy for Advanced Interdisciplinary Studies, Henan University, Kaifeng 475004, China; State Key Laboratory of Crop Stress Adaptation and Improvement, College of Life Sciences, Academy for Advanced Interdisciplinary Studies, Henan University, Kaifeng 475004, China; State Key Laboratory of Crop Stress Adaptation and Improvement, College of Life Sciences, Academy for Advanced Interdisciplinary Studies, Henan University, Kaifeng 475004, China; State Key Laboratory of Crop Stress Adaptation and Improvement, College of Life Sciences, Academy for Advanced Interdisciplinary Studies, Henan University, Kaifeng 475004, China; College of Life Sciences, Henan Normal University, Xinxiang 453007, China; State Key Laboratory of Crop Stress Adaptation and Improvement, College of Life Sciences, Academy for Advanced Interdisciplinary Studies, Henan University, Kaifeng 475004, China; State Key Laboratory of Crop Stress Adaptation and Improvement, College of Life Sciences, Academy for Advanced Interdisciplinary Studies, Henan University, Kaifeng 475004, China; Sanya Institute, Henan University, Sanya 572025, China; State Key Laboratory of Crop Stress Adaptation and Improvement, College of Life Sciences, Academy for Advanced Interdisciplinary Studies, Henan University, Kaifeng 475004, China; Plant Genetic Engineering Laboratory, School of Agriculture and Food Sciences, The University of Queensland, Brisbane, QLD 4072, Australia; State Key Laboratory of Crop Stress Adaptation and Improvement, College of Life Sciences, Academy for Advanced Interdisciplinary Studies, Henan University, Kaifeng 475004, China; Sanya Institute, Henan University, Sanya 572025, China; State Key Laboratory of Plant Cell and Chromosome Engineering, CAS Centre for Excellence in Molecular Plant Biology, Institute of Genetics and Developmental Biology, Chinese Academy of Sciences, Beijing 100101, China; State Key Laboratory of Crop Stress Adaptation and Improvement, College of Life Sciences, Academy for Advanced Interdisciplinary Studies, Henan University, Kaifeng 475004, China; Sanya Institute, Henan University, Sanya 572025, China

## Abstract

The molecular mechanisms controlling organ size during plant development ultimately influence crop yield. However, a deep understanding of these mechanisms is still lacking. UBIQUITIN-SPECIFIC PROTEASE14 (UBP14), encoded by *DA3*, is an essential factor determining organ size in Arabidopsis (*Arabidopsis thaliana*). Here, we identified two suppressors of the *da3-1* mutant phenotype, namely *SUPPRESSOR OF da3-1 1* and *2* (*SUD1* and *SUD2*), which encode the E3 ligases MOS4-ASSOCIATED COMPLEX 3A (MAC3A) and MAC3B, respectively. The *mac3a-1* and *mac3b-1* mutations partially suppressed the high ploidy level and organ size phenotypes observed in the *da3-1* mutant. Biochemical analysis showed that MAC3A and MAC3B physically interacted with and ubiquitinated UBP14/DA3 to modulate its stability. We previously reported that UBP14/DA3 acts upstream of the B-type cyclin-dependent kinase CDKB1;1 and maintains its stability to inhibit endoreduplication and cell growth. In this work, MAC3A and MAC3B were found to promote the degradation of CDKB1;1 by ubiquitinating UBP14/DA3. Genetic analysis suggests that MAC3A and MAC3B act in a common pathway with UBP14/DA3 to control endoreduplication and organ size. Thus, our findings define a regulatory module, MAC3A/MAC3B-UBP14-CDKB1;1, that plays a critical role in determining organ size and endoreduplication in Arabidopsis.

## Teaser

Analysis of 2 suppressors uncovers the molecular and genetic mechanisms by which E3 ligases MAC3A and MAC3B affect the stability of UBIQUITIN-SPECIFIC PROTEASE14 to control plant organ size.

## Introduction

Organ size is a critical factor in plant growth and development, ultimately influencing crop yield. In general, the size of an organ is determined by cell number and area, both of which are precisely established by cell proliferation and cell expansion (Dewitte and Murray 2003; Sugimoto-Shirasu and Roberts 2003; Horiguchi et al. 2006; Granier and Tardieu 2009). In plants, high ploidy levels are usually associated with cell and organ growth (Gegas et al. 2014; Xu et al. 2016; Jiang Meng, et al. 2022; Jiang Wei, et al. 2022). During organ enlargement, the endoreduplication mainly occurs in the cell differentiation process, that cells replicate their nuclear DNA without subsequently dividing, resulting in cells with higher ploidy levels (Breuer et al. 2014; Edgar et al. 2014; Lang and Schnittger 2020).

Protein ubiquitination plays a vital role in plant organ growth, with E3 ubiquitin ligase complexes such as anaphase-promoting complex/cyclosome (APC/C) playing a pivotal role in the regulation of endoreduplication (Disch et al. 2006; Heyman and De Veylder 2012). Ubiquitinated proteins can be deubiquitinated by a particular group of thiol proteases called deubiquitinating enzymes (DUBs) that are conserved among animals, plants, and fungi (Wilkinson 1997). There are 27 UBP (ubiquitin-specific protease) family members in Arabidopsis (*Arabidopsis thaliana*), grouped into 14 subfamilies (Liu et al. 2008), some of which are active in vitro (Sridhar et al. 2007). Arabidopsis’ UBIQUITIN-SPECIFIC PROTEASE14 (UBP14) belongs to a particular subfamily, encoded by the *DA3* gene (AT3G20630) (Xu et al. 2016). Two T-DNA insertion mutants of *UBP14*, *ubp14* and *titan6* (*ttn6*), display an embryo-lethal phenotype (Doelling et al. 2001; Tzafrir et al. 2002), while the *per1* mutant containing a synonymous substitution in the *UBP14* gene shows suppression of root hair elongation under phosphate deficiency conditions (Li et al. 2010). Hypomorphic *ubp14* mutant alleles have been characterized, with *da3-1* showing curly rosette leaves, larger cotyledon and petal phenotypes, and large cells due to high ploidy levels (Xu et al. 2016), while *tarani* (*tni*)*/ubp14* display aberrant embryos and decreased auxin response (Majumdar et al. 2020). UBP14/DA3 also regulates lateral root (LR) initiation by modulating auxin signaling in the pericycle and endodermis in Arabidopsis (Peng et al. 2023). UBP14/DA3 has been shown to associate with UV-B-INSENSITIVE4 (UVI4), a protein previously identified for its involvement in endoreplication, to form a UBP14/DA3–UVI4 complex which inhibits the activity of APC/C via CCS52A1 (an APC/C activator protein) (Xu et al. 2016). In turn, APC/C negatively regulates the stability of CYCLIN-DEPENDENT KINASE B1;1 (CDKB1;1) and CYCLIN A2;3 (CYCA2;3), which are direct repressors of endoreduplication (Hase et al. 2006; Boudolf et al. 2009; Heyman et al. 2011, 2017; Xu et al. 2016). Further studies have shown that the UBP14/DA3-CDKB1;1-CYCLIN-DEPENDENT KINASE G2 (CDKG2)/SUPPRESSOR OF da3-1 6 (SUD6) regulatory module triggers intracellular replication and ploidy levels, thus affecting the growth and development of cells and organs (Jiang Wei, et al. 2022). SNW/SKI-INTERACTING PROTEIN (SKIP)/SUPPRESSOR OF da3-1 3 (SUD3) acts downstream of UBP14/DA3 and UVI4 to control endoreduplication and cell growth (Jiang Meng, et al. 2022). However, the underlying mechanisms involved in the upstream regulation of UBP14 are currently unknown.

In this study, we identified 2 suppressors of *da3-1* (*SUPPRESSOR OF da3-1 1* and *2*; *SUD1* and *SUD2*) from ethyl methanesulfonate (EMS)-treated M2 populations of *da3-1*. The *sud1-1 da3-1* and *sud2-1 da3-1* double mutants suppressed the curly rosette leaves of *da3-1*. The suppressor genes *SUD1* and *SUD2* encode the U-box proteins MOS4-ASSOCIATED COMPLEX3A (MAC3A) and MAC3B, respectively. Phenotypic analysis revealed that mutations in *MAC3A* and *MAC3B* partially restored the enlarged cotyledon phenotype and reduced the ploidy level in *da3-1* plants. Additionally, MAC3A and MAC3B ubiquitinate UBP14/DA3 and negatively regulate the stability of the UBP14/DA3 protein. MAC3A and MAC3B promoted the degradation of CDKB1;1, a downstream component of APC/C, to control the onset of endoreduplication. Genetic analysis indicated that MAC3A and MAC3B act in a common pathway with UBP14/DA3 to maintain ploidy level and organ size. Our findings reveal an important genetic and molecular mechanism of the MAC3A/3B-UBP14/DA3 module-mediated control of endoreduplication and organ size in Arabidopsis.

## Results

### Multiple developmental phenotypes observed in *da3-1* mutants are partially restored in *sud1-1 da3-1* and *sud2-1 da3-1* double mutants

**Figure 1.**
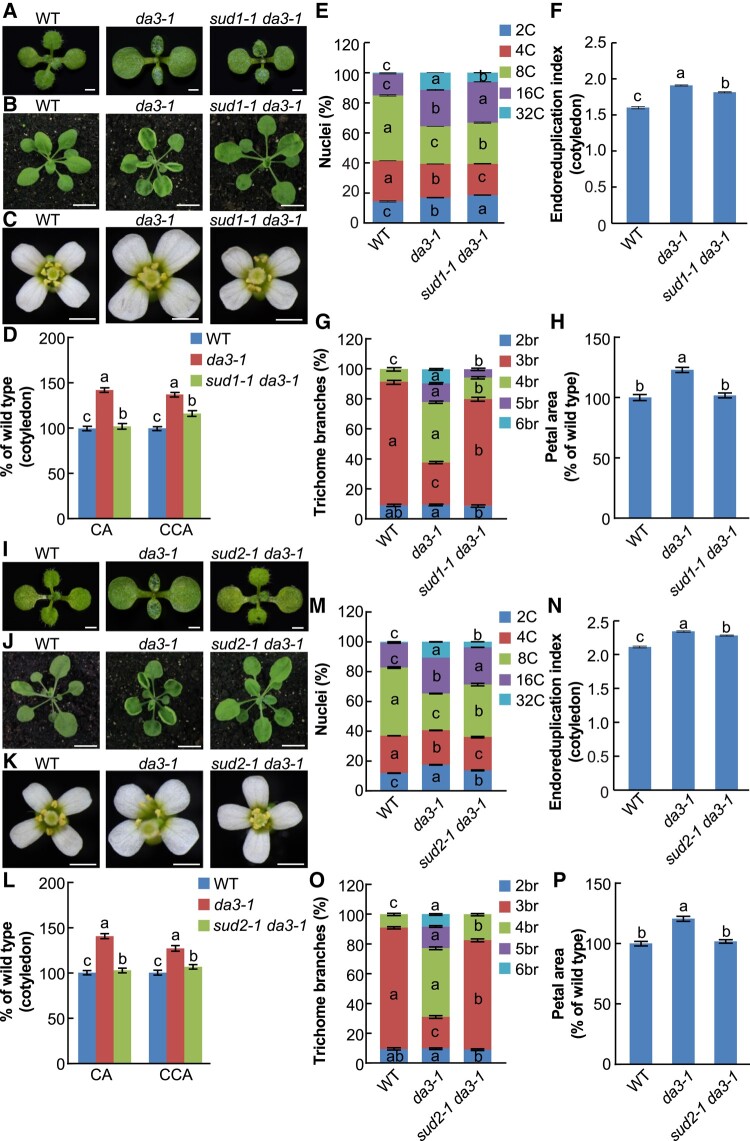
The *sud1-1* and *sud2-1* mutants partially rescue the enhanced organ growth and high ploidy level of *da3-1.***A)** to **C)** The *sud1-1* mutation suppresses the organ growth phenotypes of *da3-1*. 11-d-old seedlings **A)**, 24-d-old plants **B)**, and flowers **C)** of the genotypes WT (Col-0), *da3-1*, and *sud1-1 da3-1* (from left to right). Bars = 0.1 cm in **A)** and **C)** and 1 cm in **B)**, respectively. **D)** CA and CCA of 11-d-old WT, *da3-1*, and *sud1-1 da3-1* seedlings (*n* = 40 for CA; *n* = 30 for CCA). **E)** Nuclear DNA ploidy in cotyledons of 11 DAG WT, *da3-1*, and *sud1-1 da3-1* seedlings (*n* = 3 biological replicates). **F)** EI of 11 DAG WT, *da3-1*, and *sud1-1 da3-1* cotyledons (*n* = 3 biological replicates). **G)** Trichome branch distribution in the first pair of leaves of 15 DAG WT, *da3-1*, and *sud1-1 da3-1* seedlings (*n* = 20); br indicates the number of branches. **H)** Petal area of WT, *da3-1*, and *sud1-1 da3-1* flowers (*n* = 60). Data are mean values ± Se. Different lowercase letters indicate statistically significant differences among other groups, as determined by ANOVA and Tukey's post-hoc test (*P* < 0.05). Values in **D)** to **H)** are given as mean ± Se relative to the WT values, set at 100%. **I)** to **P)** Corresponding assays using *sud2-1* instead of *sud1-1* as described in **A)** to **H)**. DAG, days after germination. Se, standard error.

To explore the molecular mechanism used by *UBP14* in the regulation of endoreduplication and plant organ size, we produced an EMS-treated mutagenized population in a *da3-1* mutant background (partial loss function of *UBP14/DA3*) (Xu et al. 2016; Jiang Meng, et al. 2022; Jiang Wei, et al. 2022). Screening of the mutagenized population identified two suppressors of *da3-1* (*SUPPRESSOR OF da3-1 1* and *2*; *SUD1/2*), which partially restored the phenotypic developmental abnormalities observed in *da3-1* mutants to wild-type (WT) in the Columbia (Col-0) levels (Fig. 1). Phenotypic analysis showed that the *sud1-1 da3-1* mutant suppressed the enlarged cotyledons and petals, as well as the curly rosette leaves observed in *da3-1* (Fig. 1, A to C). The cotyledon area (CA) in the *sud1-1 da3-1* double mutant was significantly smaller than in *da3-1* (Fig. 1D). The cellular analysis of *da3-1* and *sud1-1 da3-1* cotyledons showed that the cotyledon palisade cell area of *da3-1* was reduced in the *sud1-1 da3-1* double mutant (Fig. 1D). Since the cotyledon cell area (CCA) is often associated with changes in ploidy level, we performed a flow cytometry analysis of nuclear DNA. We observed that the number of 32C cells in *sud1-1 da3-1* cotyledons was significantly reduced compared with *da3-1* (Fig. 1E). Furthermore, the endoreduplication index (EI) of *sud1-1 da3-1* mutants is lower than *da3-1* but higher than WT plants (Fig. 1F). Then, we counted trichome branch numbers from the first pair of leaves from WT, *da3-1*, and *sud1-1 da3-1*. The number of trichomes with 4, 5, and 6 branches was significantly lower in *sud1-1 da3-1* than in *da3-1* (Fig. 1G). Moreover, the enlarged petal area observed in the *da3-1* mutant was also suppressed by the mutation of *sud1-1* (Fig. 1H). Meanwhile, the *sud2-1 da3-1* mutants showed a similar phenotype with *sud1-1 da3-1* (Fig. 1, I to P). Collectively, these data indicate that the *sud1-1* and *sud2-1* mutations partially suppress the high ploidy, cell, and organ growth phenotypes observed in *da3-1* mutants.

### Identification of *SUD1* and *SUD2*

**Figure 2.**
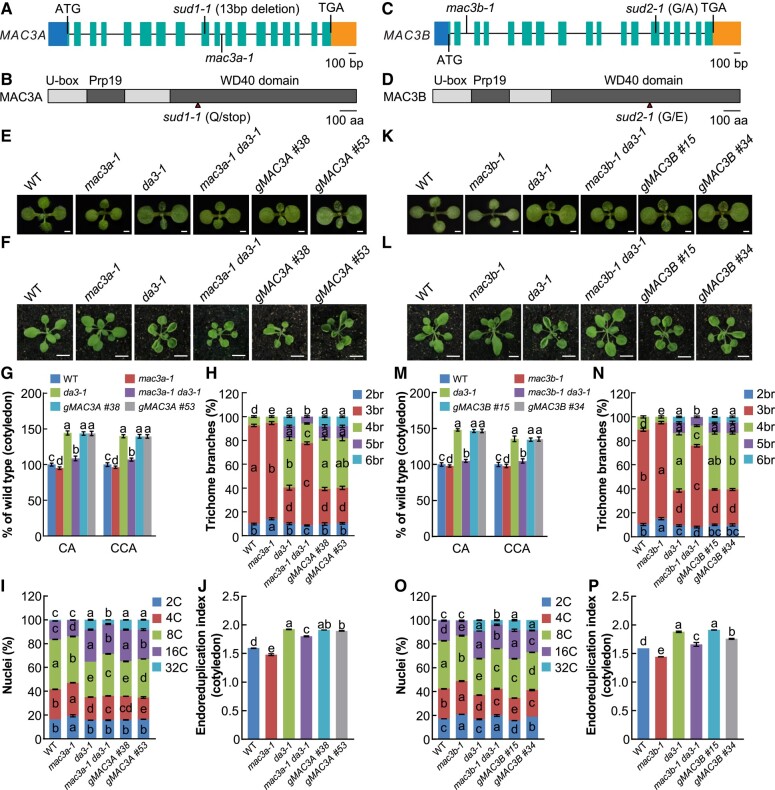
Identification and cloning of *SUD1* (*MAC3A*) and *SUD2* (*MAC3B*) genes. **A)***MAC3A* gene structure. The start codon (ATG) and the stop codon (TGA) are indicated. 5'UTR is indicated in front of ATG, while 3'UTR is indicated in the behind of TGA. Exons are represented as green boxes, while lines represent introns. The *sud1-1* mutation consists of a 13 bp deletion, and the position of the T-DNA insertion in the *mac3a-1* mutant is indicated. **B)** Schematic of MAC3A protein. The mutations in *sud1-1* result in early terminations. **C)***MAC3B* gene structure. The start codon (ATG) and the stop codon (TGA) are indicated. 5'UTR is indicated in front of ATG, while 3'UTR is indicated in the behind of TGA. Exons are represented as green boxes, while lines represent introns. The *sud2-1* mutation consists of a point mutation of G to T, and the position of the T-DNA insertion in the *mac3b-1* mutant is indicated. **D)** Schematic of MAC3B protein. The mutations in *sud2-1* result in a point mutation. **E)** to **F)** Phenotypes of WT (Col-0), *mac3a-1*, *da3-1*, *mac3a-1 da3-1*, *gMAC3A #38*, and *gMAC3A #53* plants. The *gMAC3A* transgenic plants contain a fragment including the promoter and coding region of the native *MAC3A* gene in a *mac3a-1 da3-1* background and complement the organ growth phenotypes of the *mac3a-1 da3-1* double mutant to resemble single *da3-1* mutants of 11-d-old seedlings **E)** and 24-d-old plants **F)**. Bars = 0.1 cm in **E)** and 1 cm in **F)**, respectively. **G)** CA and CCA of 11-d-old plants from the genotypes described in **E)** (*n* = 40 for CA; *n* = 30 for CCA). **H)** Trichome branch distribution in the first pair of leaves of the genotypes described in **E)** at 15 DAG (*n* = 20). br indicates the number of branches. **I)** Nuclear DNA ploidy in cotyledons of the genotypes described in **E)** seedlings at 11 DAG (*n* = 3 biological replicates). **J)** EI of cotyledons from the genotypes described in **E)** seedlings at 11 DAG (*n* = 3 biological replicates). Data are mean values ± Se. Different lowercase letters indicate statistically significant differences among other groups, as determined by ANOVA and Tukey’s post-hoc test (*P* < 0.05). Se, standard error. Values in **G)** to **I)** are given as mean ± Se relative to the WT values, set at 100%. **K)** to **P)** Corresponding assays were done for WT, *mac3b-1*, *da3-1*, *mac3b-1, da3-1*, *gMAC3B #15*, and *gMAC3B #34* as in **E)** to **J)**. DAG, days after germination.

The causal suppressor genes for the *sud1-1* and *sud2-1* mutations were identified as *MAC3A* and *MAC3B*, respectively, using MutMap and Sanger sequencing approaches (Supplemental Tables S1 and S2). The *sud1-1* mutation contains a 13 nucleotide deletion in the coding region of *MAC3A*, leading to a truncated protein with 256 amino acids (Supplemental Fig. S1A; Fig. 2, A and B). The *sud2-1* mutation contains a G to A substitution at position 1,106 in the *MAC3B* coding sequence (CDS), resulting in a glycine to glutamic acid change at position 369 of the protein (Supplemental Fig. S1B; Fig. 2, C and D). Bioinformatic analysis showed that Arabidopsis MAC3A and MAC3B proteins share 82% sequence identity (Supplemental Fig. S2A). MAC3A and MAC3B are 2 homologous U-box domain E3 ubiquitin ligases highly similar to the E3 ubiquitin ligase Prp19 in *Schizosaccharomyces pombe* and humans (Monaghan et al. 2009). MAC3A and MAC3B are conserved in the plant kingdom (Supplemental Fig. S2B) and play essential roles in the accumulation of miRNAs via the DICER-LIKE1 (DCL1) complex, ABA response, plant immunity, and development (Zhang et al. 2008; Monaghan et al. 2009; Jia et al. 2017; Li et al. 2018). To confirm the identity of the *MAC3A* and *MAC3B* genes as the causal agents for the *sud1-1* and *sud2-1* mutations, we generated double mutants by crossing the *da3-1* mutant with the T-DNA insertion mutants *mac3a-1* (Salk_089300) and *mac3b-1* (Salk_130035), respectively (Fig. 2, A to D). The *mac3a-1 da3-1* double mutant showed similar phenotypes to those observed in *sud1-1 da3-1* mutant in which partial restoration of the *da3-1* mutant phenotypes was observed, including cotyledon and petal size, CCA, trichome branching, ploidy, and EI (Fig. 2, E to J; Supplemental Fig. S3). Likewise, the *mac3b-1 da3-1* double mutant phenocopied the *sud2-1 da3-1* mutants (Fig. 2, K to P). These data support the identity of *MAC3A* and *MAC3B* as *SUD1* and *SUD2*, respectively. Compared with WT plants, individual *mac3a-1* and *mac3b-1* mutants showed statistically significant differences in smaller CA, reduced number of 16C cells, lower EI, and reduced number of trichomes with 4 branches.

To provide further evidence for the identity of *SUD1/2*, we performed complementation analysis by introducing a genomic fragment containing the WT *MAC3A* or *MAC3B* genes driven by their native promoters into the *mac3a-1 da3-1* or *mac3b-1 da3-1* background, respectively (named *gMAC3A* and *gMAC3B*). Analysis of 2 independent lines for each of the complementation genotypes showed that *gMAC3A* and *gMAC3B* lines restored the phenotypes of *mac3a-1 da3-1* and *mac3b-1 da3-1* double mutants to those observed in *da3-1* mutants, respectively, including CA, cell area, trichome branch number, ploidy, and EI (Fig. 2, E to P). Taken together, our data strongly support the role of *MAC3A* and *MAC3B* as suppressors of *UBP14/DA3*.

### *MAC3A* and *MAC3B* positively regulate organ size in Arabidopsis

**Figure 3.**
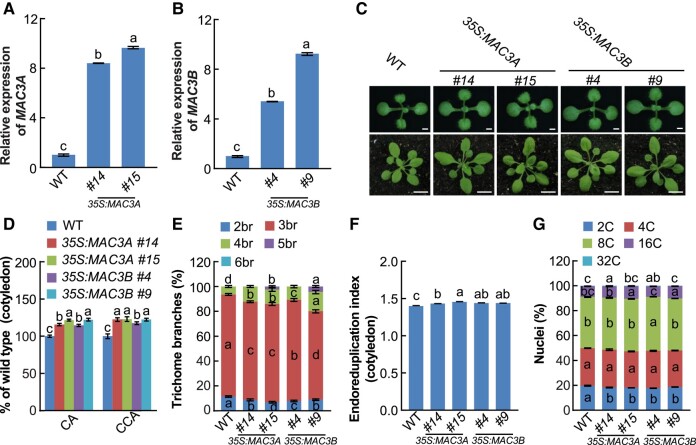
MAC3A and MAC3B positively regulate endoreduplication. **A)** Relative *MAC3A* transcript levels in WT (Col-0), *35S:MAC3A #14*, and *35S:MAC3A #15* OE transgenic plants. **B)** Relative *MAC3B* transcript levels in WT, *35S:MAC3B #4*, and *35S:MAC3B #9* OE transgenic plants. **C)** The *35S:MAC3A* and *35S:MAC3B* OE transgenic lines exhibit enlarged organ phenotypes compared with WT. 10-d-old seedlings (upper panel) and 24-d-old plants (lower panel) of WT, *35S:MAC3A #14*, *35S:MAC3A #15*, *35S:MAC3B #4*, and *35S:MAC3B #9* (from left to right). Scale bars = 0.1 cm (upper panel) and 1 cm (lower panel), respectively. **D)** CA and CCA of 10-d-old WT, *35S:MAC3A #14*, *35S:MAC3A #15*, *35S:MAC3B #4*, and *35S:MAC3B #9* seedlings (from left to right) (*n* = 40 for CA; *n* = 30 for CCA). **E)** Trichome branch distribution in the first pair of leaves of 15 DAG WT, *35S:MAC3A #14*, *35S:MAC3A #15*, *35S:MAC3B #4*, and *35S:MAC3B #9* seedlings (*n* = 20). br indicates the number of branches. **F)** EI of 10 DAG WT, *35S:MAC3A #14*, *35S:MAC3A #15*, *35S:MAC3B #4*, and *35S:MAC3B #9* cotyledons (*n* = 3 biological replicates). **G)** Nuclear DNA ploidy in cotyledons of 10 DAG WT, *35S:MAC3A #14*, *35S:MAC3A #15*, *35S:MAC3B #4*, and *35S:MAC3B #9* seedlings (*n* = 3 biological replicates). Data are mean values ± Se. Se, standard error. Different lowercase letters indicate statistically significant differences among different groups, as determined by ANOVA and Tukey's post-hoc test (*P* < 0.05). Values in **A)**, **B)**, **D)**, and **F)** are given as mean ± Se relative to the WT values, set at 100%. DAG, days after germination.

To better understand the role of MAC3A and MAC3B in organ size control, we generated overexpression (OE) lines by transforming WT plants with the *MAC3A* and *MAC3B* genes driven by the *CaMV 35S* promoter. Two independent transgenic lines for each genotype (*35S:MAC3A #14/#15* and *35S:MAC3B #4/#9*) with elevated transcript levels (Fig. 3, A and B) were further characterized. All four overexpressing transgenic lines exhibited increased cotyledon organ size and cell area, compared with WT (Fig. 3, C and D). Differences in trichome branch numbers were also observed, with a larger number of trichomes containing 4 and 5 branches observed in overexpressing lines (Fig. 3E). Further, the EI of all OE lines was higher than WT controls (Fig. 3F), while analysis of nuclear DNA content showed that the number of 16C and 32C cells in *35S:MAC3A #15* and *35S:MAC3B #9* cotyledons was higher than WT (Fig. 3G). Collectively, the phenotypes of the single *mac3a-1* and *mac3b-1* mutants and overexpressing lines suggest that MAC3A and MAC3B play positive roles in regulating organ size in an organ-dependent manner in Arabidopsis.

### *MAC3A* and *MAC3B* genetically interact with *UBP14* to control endoreduplication and organ size

Plant organ size is generally determined by cell area and number (Horiguchi et al. 2006). Previously, UBP14/DA3 was found to interact with UVI4 to suppress endoreduplication, further negatively regulating organ size (Xu et al. 2016). Considering the *da3-1* cotyledons contained larger cells than WT cotyledons, we measured CA and CCA in WT, *da3-1*, *mac3a-1*, *mac3a-1 da3-1*, *mac3b-1*, and *mac3b-1 da3-1* seedlings at 11 days after germination (DAG). Palisade cells in *mac3a-1 da3-1* and *mac3b-1 da3-1* cotyledons were smaller than those in *da3-1* cotyledons, and CA in *mac3a-1 da3-1* and *mac3b-1 da3-1* were smaller than in *da3-1* (Fig. 2, G and M). We then tested whether the CCA phenotype in *mac3a-1 da3-1* and *mac3b-1 da3-1* cotyledons are associated with changes in ploidy level. The cell ploidy ratio of 16C to 32C levels in *da3-1* was much higher than in WT, while *mac3a-1 da3-1*, and *mac3b-1 da3-1* mutants showed partial restoration of ploidy numbers of *da3-1* mutant (Fig. 2, I and O). High ploidy level is known to be linked to an increased number of trichome branches in Arabidopsis (Xu et al. 2016); thus, we measured the number of trichome branches in WT, *da3-1*, *mac3a-1 da3-1*, and *mac3b-1 da3-1* mutants. The numbers of trichomes with 4, 5, and 6 branches were lower in *mac3a-1 da3-1* and *mac3b-1 da3-1* leaves than in *da3-1* leaves, suggesting that MAC3A/3B and DA3 act in a common pathway to control the number of trichome branches (Fig. 2, H and N).

Additionally, the *mac3a-1 da3-1* double mutant was crossed with the *mac3b-1* mutant to produce triple mutant *mac3a-1 mac3b-1 da3-1*, suppressing the CA of *da3-1* (Supplemental Fig. S4). The plant morphology of *mac3a-1 mac3b-1 da3-1* triple mutant was nearly restored to WT (Supplemental Fig. S4). Collectively, these data suggest that *MAC3A* and *MAC3B* genetically interact with *UBP14/DA3* to regulate endoreduplication and organ size.

### MAC3A and MAC3B physically associate with UBP14/DA3

**Figure 4.**
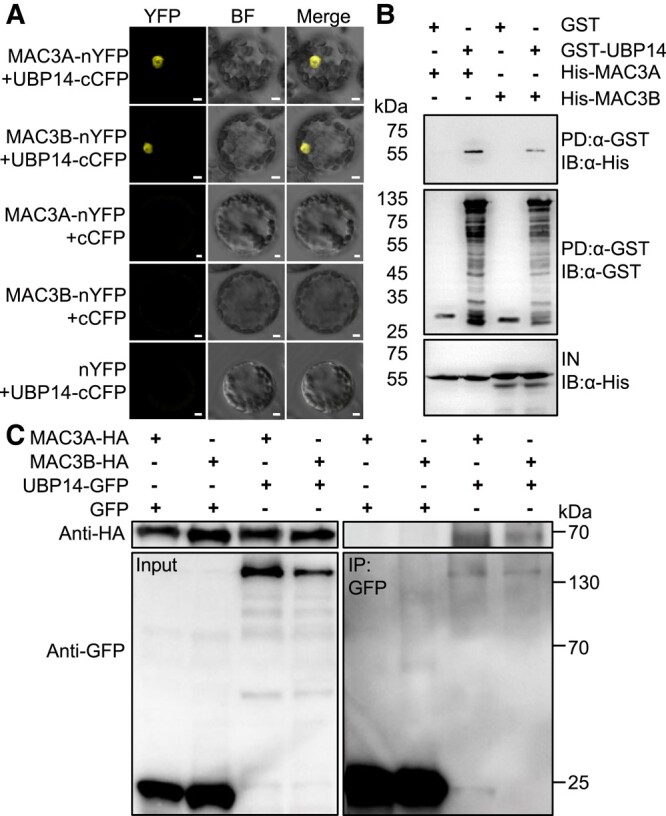
MAC3A and MAC3B physically interact with UBP14. **A)** BiFC assays showing the interaction between MAC3A/3B and UBP14 in the nucleus of leaf mesophyll protoplasts. Protoplasts were prepared from 1-mo-old seedlings and transformed with 10 *μ*g plasmid DNA, as indicated in the figure. As negative controls, MAC3A-nYFP + cCFP (cyan fluorescent protein), MAC3B-nYFP + cCFP, and nYFP + UBP14-cCFP were used. YFP fluorescence was observed with a Zeiss LSM980 laser confocal microscope. Bars = 5 *μ*m. BF indicates bright field. Bars = 5 *μ*m. 1-mp-old, 1-month-old. **B)** GST pull-down assays showing the interaction between MAC3A/3B and UBP14. UBP14 fused to GST tag was used to pull down (PD) His-MAC3A and His-MAC3B, respectively. Proteins in the upper and lower panels were detected by immunoblotting (IB) using an anti-α-His antibody, and those in the middle panel were detected by IB using an anti-α-GST antibody. IN represents input. **C)** CoIP analysis showing the interaction between MAC3A/3B and UBP14 in vivo. Plasmids *Pro35S:MAC3A-HA*, *Pro35S:MAC3B-HA*, *Pro35S:UBP14-GFP*, and *Pro35S:GFP* were transiently expressed in *N. benthamiana* leaves in different combinations using *Agrobacterium-*mediated infiltration. Total proteins were extracted and immunoprecipitated (IP) with GFP-Trap-A, and the immunoblot was probed with anti-GFP and anti-HA antibodies, respectively.

The genetic analyses indicate that MAC3A and MAC3B function in a common genetic pathway with UBP14/DA3 to regulate endoreduplication and organ size in Arabidopsis. In addition, transient expression of MAC3A-green fluorescent protein (GFP), MAC3B-GFP, and UBP14/DA3–GFP fusion proteins in Arabidopsis protoplasts show that all of them colocalize to the nucleus (Supplemental Fig. S5), as previously reported (Monaghan et al. 2009; Xu et al. 2016). We therefore queried whether MAC3A and MAC3B can physically interact with UBP14/DA3. In an initial approach to determine the possible interaction of MAC3A and MAC3B with UBP14/DA3, we conducted bimolecular fluorescence complementation (BiFC) assays. When *MAC3A-nYFP* (yellow fluorescent protein) or *MAC3B-nYFP* was transiently coexpressed with *UBP14-cYFP* in Arabidopsis protoplast cells, we detected strong YFP signals in nuclei. In contrast, no signal was detected in negative controls (Fig. 4A), suggesting that MAC3A and MAC3B interact with UBP14/DA3 in the nucleus. Glutathione S-transferase (GST) pull-down assays supported the BiFC results, showing the interaction between UBP14/DA3 and either MAC3A or MAC3B (Fig. 4B). Finally, we performed co-immunoprecipitation (CoIP) assays to support the interaction results further. For this purpose, we transiently coexpressed MAC3A or MAC3B with UBP14/DA3 in the leaves of *Nicotiana benthamiana*. Total proteins were extracted and incubated with GFP-Trap-A agarose beads. MAC3A or MAC3B was detected in the immunoprecipitated UBP14/DA3–GFP complexes but not in the immunoprecipitated GFP complexes (Fig. 4C). These results demonstrate that UBP14/DA3 can physically interact with both MAC3A and MAC3B.

### MAC3A and MAC3B ubiquitinate UBP14 and regulate its stability

**Figure 5.**
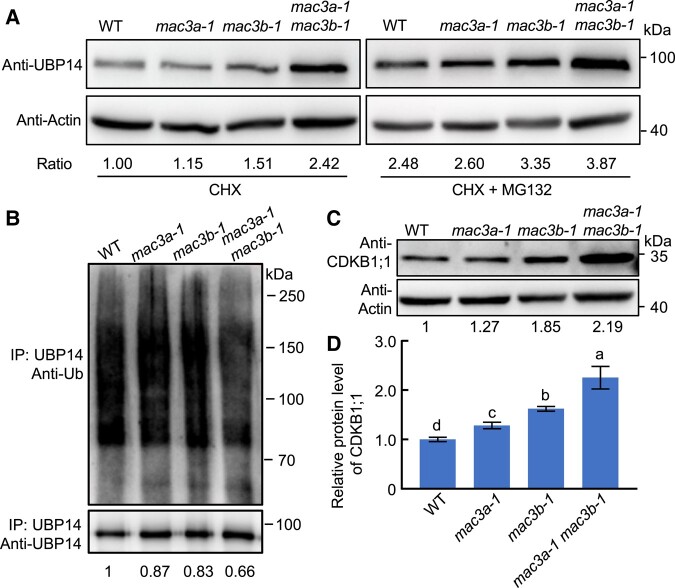
MAC3A and MAC3B can ubiquitinate UBP14 and regulate its stability. **A)** UBP14 protein levels were detected by Western blot in WT (Col-0), *mac3a-1*, *mac3b-1*, and *mac3a-1 mac3b-1* plants. 11-d-old WT, *mac3a-1*, *mac3b-1*, and *mac3a-1 mac3b-1* seedlings were pretreated with 100 *μ*M CHX in the absence or presence of 50 *μ*M MG132 for 3 h. Whole seedlings were harvested, and total protein was extracted. An anti–UBP14-specific antibody was used to detect UBP14. Actin was used as a loading control. Ratio values of UBP14 protein levels to Actin levels were given as mean ± Sd (*n* = 3 biological replicates) relative to the values of WT under CHX treatment (artificially set at 1.0). Sd, standard deviation. **B)** UBP14 ubiquitination analysis. Total proteins were extracted from 11-d-old plants of WT, *mac3a-1*, and *mac3a-1 mac3b-1* double mutant plants before being immunoprecipitated (IP) using UBP14 antibodies with the Protein A/G Magnetic Beads. The proteins were then separated by electrophoresis and subjected to IB with either anti-UBP14 or anti-ubiquitination (Ub) antibodies. Ubiquitinated UBP14 protein levels were expressed relative to immunoprecipitated UBP14. Relative protein levels of ubiquitinated UBP14 were calculated relative to the value of WT (artificially set at 1.0). **C)** CDKB1;1 protein level was detected by Western blotting in WT, *mac3a-1*, *mac3b-1*, and *mac3a-1 mac3b-1* plants. 11-d-old seedlings were harvested, and total protein was extracted. Anti-CDKB1;1 specific antibody was used to detect protein levels. Actin was used as a loading control. CDKB1;1 relative protein level was calculated relative to Actin. Values are given as mean ± Sd (*n* = 3 biological replicates) relative to the value for WT (artificially set at 1.0). **D)** CDKB1;1 protein levels of WT, *mac3a-1*, *mac3b-1*, and *mac3a-1 mac3b-1* double mutant plants relative to WT **C)**. Values are given as mean ± Sd (*n* = 3 biological replicates) relative to the value for WT, set at 100% **D)**. Sd, standard deviation. Different lowercase letters indicate statistically significant differences among other groups, as determined by ANOVA and Tukey's post-hoc test (*P* < 0.05).

MAC3A and MAC3B share a high degree of sequence identity with Prp19 and were reported to possess ubiquitin ligase activities (Li et al. 2008; Monaghan et al. 2009; Jia et al. 2017). Therefore, we hypothesized that MAC3A/MAC3B can ubiquitinate UBP14/DA3 protein to control its stability. To test this hypothesis, UBP14/DA3 protein levels were measured in WT, *mac3a-1*, *mac3b-1*, and *mac3a-1 mac3b-1* mutants using an antibody directed against the native UBP14/DA3 in plants treated with cycloheximide (CHX; an mRNA translation inhibitor to block de novo protein synthesis) in the presence or absence of the proteasome inhibitor MG132. Under the treatment of the de novo protein synthesis inhibitor CHX, the UBP14 protein is fixed at its initial levels. As shown in Fig. 5A (left panel), UBP14/DA3 was detected in WT, *mac3a-1*, and *mac3b-1*, but its protein level was increased in *mac3a-1 mac3b-1* compared with WT (see quantification of band intensities). This result suggests that MAC3A and MAC3B jointly destabilize UBP14/DA3. The seedlings of WT, *mac3a-1*, *mac3b-1*, and *mac3a-1 mac3b-1* mutants were treated with MG132 (a proteasome inhibitor), and it showed that the destabilization of UBP14/DA3 mediated by both MAC3A and MAC3B was reversed by MG132 to some extent, suggesting that MAC3A and MAC3B downregulated the UBP14/DA3 protein through the ubiquitin proteasome pathway (Fig. 5A, right panel). These assays were repeated 3 more times with very similar results (Supplemental Fig. S6, A and B). Considering the potential ubiquitin ligase activity of MAC3A and MAC3B, we detected UBP14/DA3 ubiquitination levels of *mac3a-1*, *mac3b-1*, and *mac3a-1 mac3b-1* mutants. The *mac3a-1 mac3b-1* double mutant decreased the ubiquitination of UBP14/DA3, compared with the WT, as shown in Fig. 5B. These results demonstrated that MAC3A and MAC3B could increase the UBP14 ubiquitination and decrease the abundance of UBP14 protein in Arabidopsis.

Considering that UBP14/DA3–UVI4 complex modulates the stability of CDKB1;1 (Heyman et al. 2011; Xu et al. 2016), it is understandable that MAC3A and MAC3B impact the degradation through ubiquitination of UBP14. In this study, western blot analysis of total protein extracts from WT, *mac3a-1*, *mac3b-1*, and *mac3a-1 mac3b-1* plants was conducted. The results indicated that CDKB1;1 protein levels were increased in *mac3a-1 mac3b-1* mutants compared with WT (Fig. 5, C and D; Supplemental Fig. S7). Taken together, MAC3A and MAC3B were found to promote the degradation of CDKB1;1 through ubiquitination of UBP14/DA3, resulting in a high level of endopolyploidy linked to organ enlargement.

**Figure 6.**
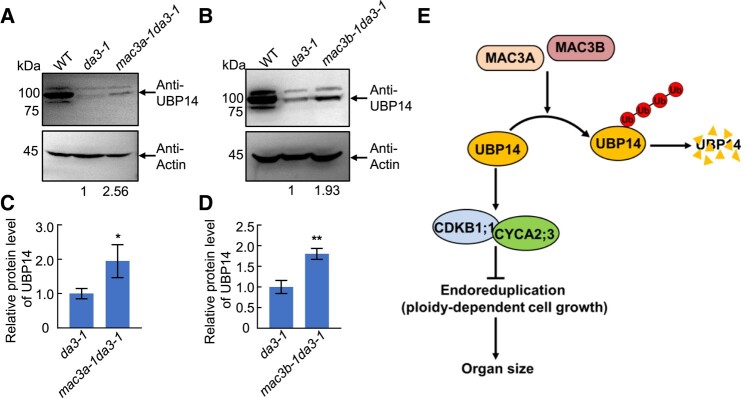
*MAC3A* and *MAC3B* act downstream of *UBP14* to regulate endoreduplication and cell and organ growth. **A)** and **B)** UBP14 protein abundance was detected by Western blotting in 11 DAG WT (Col-0), *da3-1*, and either *mac3a-1 da3-1***A)** or *mac3b-1 da3-1***B)** seedlings. Total proteins were extracted from seedlings and separated by electrophoresis. Anti–UBP14-specific antibodies were used to detect UBP14 in Western blots. Actin was used as a loading control to calculate the relative abundance of UBP14. **C)** UBP14 protein levels in *mac3a-1 da3-1* seedlings expressed relative to *da3-1***A)**. Values are given as mean ± Sd (*n* = 3 biological replicates) relative to the value for *da3-1*, set at 100% **C)**. Sd, standard deviation. *P < 0.05 (Student's *t* test). **D)** UBP14 protein levels in *mac3b-1 da3-1* expressed relative to *da3-1***B)**. Values are given as mean ± Sd (*n* = 3 biological replicates) relative to the value for *da3-1*, set at 100% **D)**. **P < 0.01 (Student's *t* test). Sd, standard deviation. **E)** Working model for MAC3A/MAC3B involvement in endoreduplication and cell and organ growth. MAC3A and MAC3B physically interact with UBP14 and ubiquitinate UBP14. Thereby, MAC3A and MAC3B negatively regulate the protein abundance of UBP14. Degradation of UBP14 modulates the quantity of CDKB1;1 and CYCA2;3 to control endoreduplication and cell growth, thereby influencing organ size. Blunt arrows indicate inhibition. Ub, ubiquitin.

We have previously reported that the *da3-1* mutation results in a partial loss of function mutant of UBP14, and the UBP14*^da3–1^* mutation disrupts the deubiquitination activity of UBP14 (Xu et al. 2016). In the *da3-1* mutant, a low level of UBP14 protein was still detected (Fig. 6A). We observed that *mac3a-1* and *mac3b-1* partially suppress the phenotype of *da3-1* by controlling endoreduplication. Moreover, our results demonstrate that MAC3A/3B ubiquitinates UBP14 in vivo and influences DA3 protein abundance. We hypothesized that the decreased ubiquitination levels of UBP14 in *mac3a-1* and *mac3b-1* caused the observed protein abundances of UBP14 in *mac3a-1 da3-1* and *mac3b-1 da3-1* double mutants. Therefore, *mac3a-1* and *mac3b-1* can suppress endoreduplication phenotypes of *da3-1*. Consistently, we identified the UBP14 protein levels in *mac3a-1 da3-1* and *mac3b-1 da3-1* double mutants, and both the mutations in MAC3A and MAC3B can partially rescue UBP14 protein levels in *da3-1* mutants (Fig. 6, A to D; Supplemental Fig. S8). These results support that *mac3a-1* and *mac3b-1* partially block the *da3-1* phenotype by partially enhancing DA3 protein abundance.

## Discussion

Plant organ size, essential for crop yield, is determined by cell number and area via cell expansion and proliferation (Granier and Tardieu 2009). Chromosome ploidy, modulated by endoreduplication, has been found to play a vital role in the control of organ size and is determined by the catalytic subunits of cyclin-dependent kinases (CDK) and cyclins (CYC) by triggering the initiation of nuclear replication. UBP14/DA3 and UVI4 act upstream of CDKB1;1 and CYCA2;3 to regulate endoreduplication and organ size (Xu et al. 2016). Some downstream components of UBP14/DA3 in the control of endoreduplication and cell and organ growth, such as SUD6 and SUD3, are known (Jiang Meng, et al. 2022; Jiang Wei, et al. 2022). However, the underlying mechanisms involved in the upstream regulation of UBP14/DA3 are largely unknown. In this study, we provide evidence that the MAC3A/MAC3B-UBP14/DA3 regulatory module controls endoreduplication and organ size in Arabidopsis.

Two independent suppressors of the *da3-1* mutant originating from mutations in *MAC3A* and *MAC3B* partially restore the increased organ sizes observed in *da3-1* mutants by decreasing ploidy levels. UBP14/DA3 works with UVI4 to negatively regulate the APC/C ubiquitin ligase, thus affecting cell ploidy and endoreduplication (Xu et al. 2016). Our findings suggest that MAC3A and MAC3B genetically antagonize the function of UBP14/DA3, possibly by affecting UBP14/DA3 stability via ubiquitination. Biochemical evidence identifying *MAC3A* and *MAC3B* as U-box E3 ubiquitin ligases (Monaghan et al. 2009) supports our hypothesis. Further support comes from our findings that MAC3A and MAC3B physically interact with UBP14/DA3 in vitro and in vivo and ubiquitinate the UBP14/DA3 protein. It is essential to keep in mind that the *da3-1* mutation only causes a partial loss of function in UBP14, producing a truncated protein (Xu et al. 2016). Thus, according to our hypothesis, MAC3A and MAC3B can negatively regulate the UBP14/DA3 protein levels via ubiquitination, and mutations in *MAC3A* and *MAC3B* could result in increased UBP14/DA3 protein levels as we show in either a WT background (Fig. 5A; Supplemental Fig. S6) or a *da3-1* mutant background (Fig. 6, A to D; Supplemental Fig. S8).

It is well known that endoreduplication is precisely regulated by CYCA2;3 and CDKB1;1, 2 downstream components of the APC/C ubiquitin ligase pathway, to control cell growth (Xu et al. 2016). UBP14/DA3 was shown to negatively regulate APC/C and increase CDKB1;1 protein level to control endoreduplication and cell and organ growth (Xu et al. 2016). Our work shows that CDKB1;1 level was increased in the *mac3a-1 mac3b-1* double mutant, suggesting that MAC3A and MAC3B negatively regulate CDKB1;1, to positively regulate endoreduplication. Given that *mac3a-1 mac3b-1* mutants exhibited smaller organ sizes than WT, MAC3A, and MAC3B seem to jointly play positive roles in regulating organ size, whereas UBP14/DA3 plays a negative role. Our results demonstrate that the DA3 protein abundances *mac3a-1 da3-1* and *mac3b-1 da3-1* double mutants are elevated but lower than that in WT (Fig. 6, A to D) and partially suppressed the increased cell area and organ growth observed in *da3-1* mutants, indicating that MAC3A and MAC3B act in a common pathway with UBP14/DA3. Further, MAC3A and MAC3B were found to interact with UBP14/DA3 and determined UBP14/DA3 protein levels via ubiquitination, which suggests that MAC3A and MAC3B act as upstream components of the pathway with UBP14/DA3. Some ubiquitination modifications of UBP14 were detected in *mac3a-1 mac3b-1* double mutant (Fig. 5B), which may imply the presence of other factors working together to regulate the endoreduplication and organ size in Arabidopsis. Moreover, the protein stability of CDKB1;1 was also negatively affected by MAC3A and MAC3B, indicating that MAC3A and MAC3B regulated UBP14/DA3 to control the endoreduplication and organ size via the already established UBP14-CDKB1;1-CYCA2;3 pathway (Xu et al. 2016).

Collectively, we propose a model in which MAC3A and MAC3B regulate the stability of UBP14/DA3 via ubiquitination and contribute to maintaining the balance of endoreduplication level to fine-tune the control of organ size, by indirectly regulating CDKB1;1 protein abundance (Fig. 6E). Identification of the MAC3A/MAC3B-UBP14 regulatory module expands our knowledge of endoreduplication control and organ development, which could be harnessed in future breeding efforts to achieve crop yield increases.

## Materials and methods

### Plant materials and growth conditions

All Arabidopsis (*A. thaliana*) mutants used in this study were in the WT (Col-0) background. The *sud1-1 da3-1* and *sud2-1 da3-1* suppressors were obtained from an M_2_ population derived from *da3-1* treated with the chemical mutagen EMS. The T-DNA insertion lines *mac3a-1* (Salk_089300) and *mac3b-1* (Salk_130035) were obtained from the Nottingham Arabidopsis Stock Centre and the Arabidopsis Biological Resource Center (ABRC; The Ohio State University). The seeds were sterilized with 10% (*v*/*v*) NaClO for 10 min, washed 5 times with sterile water, and sown on 1/2 MS medium (Murashige and Skoog 1962). The seeds were stratified by placing the plates at 4°C for 3 d before they were transferred to the light. Plants were grown at 22°C under long-day (16 h light/8 h dark) conditions (Li et al. 2022; Zhou et al. 2022).

### Morphological and cellular analysis

Cotyledons and petals were photographed under an OLYMPUS microscope (OLYMPUS SZX2-TR30) with an OLYMPUS camera (DF PLAPO). The first pair of leaves of 15-d-old seedlings was used to measure trichome branches using an OLYMPUS microscope (OLYMPUS SZX2-TR30) with an OLYMPUS camera (DF PLAPO).

For cell area, cotyledons were cleared in a clearing solution (24 g of chloral hydrate, 9 mL of ultrapure water, and 3 mL of glycerol) and photographed under a differential interference contrast microscope (Yuan et al. 2020; Jiang Wei, et al. 2022). The areas of cotyledons, petals, and cotyledons cells were measured using ImageJ software after photographing. Statistical analysis was performed by ANOVA and Tukey's post-hoc test (*P* < 0.05).

### Flow cytometry assays

Cotyledons were chopped in GS buffer (45 mM MgCl_2_, 20 mM MOPS, 30 mM sodium citrate, 0.1% Triton X-100), and nuclei were obtained by passing the slurry through a sieve (38 *μ*m^2^ mesh). Nuclei were stained with 10 *μ*M DAPI, and the nuclear DNA content was analyzed with a flow cytometer (BD FACSAria II). The values were analyzed against relative fluorescence intensities of the WT. The experiment was performed in 3 biological replicates with 3 measurements for each biological sample. EI was calculated as described previously (Jiang Meng, et al. 2022). Statistical analysis was performed by ANOVA and Tukey’s post-hoc test (*P* < 0.05).

### Identification of the *SUD1* and *SUD2* gene

The MutMap approach was conducted to identify the *sud1-1* mutations using the F2 population of a cross between *sud1-1 da3-1* and *da3-1*. In the F2 population, the separation ratio was 3:1, suggesting that a single recessive mutation determined the phenotypes of *sud1-1*. We extracted DNA from 72 plants in the F2 population that exhibited the *sud1-1 da3-1* phenotypes and mixed equal amounts of DNA from these plants for whole-genome sequencing. DNA from *da3-1* was sequenced as a control. We detected 33,036 single nucleotide polymorphisms (SNPs) and 7,267 insertions and deletions (INDELs) between the pooled F2 samples and *da3-1*. Given that all mutant plants in the F2 population were presumed to possess the causative SNP/INDEL, the SNP/INDEL ratio for this causative mutation in bulk F2 plants should be 1. In total, 1 INDEL and 2 SNPs had an SNP/INDEL index = 1 (Supplemental Table S1). Only INDEL71 was identified in exons (Supplemental Table S1). We further developed the Cleaved Amplified Polymorphic Sequences (CAPS) marker based on the INDEL71 mutation and found INDEL71 was cosegregated with the *sud1-1 da3-1* phenotypes. The INDEL71 contained a 13 bp deletion in *sud1-1* in the gene *AT1G04510*, resulting in a premature stop codon. These results suggested that *AT1G04510* was the candidate gene for *SUD1*.

The *sud2-1* mutation was identified utilizing a similar method to that of *sud1-1*. In total, 3 SNPs had an SNP/INDEL index = 1 (Supplemental Table S2). Among these, only SNP11677 occurred in exons. SNP11677 involved a G to A substitution in the *AT2G33340* gene, causing a glycine to glutamic acid change. We developed a dCAPS marker based on this mutation and determined that SNP11677 cosegregated with the *sud2-1 da3-1* phenotypes. These results suggest that *AT2G33340* is the candidate gene of *SUD2*.

### Phylogenetic analysis

MAC3A and MAC3B protein sequence alignments were carried out using GeneDoc software. MAC3A, MAC3B, and Prp19 homologs protein sequences from multiple species were selected from the UniProt database (https://www.uniprot.org/), and the maximum likelihood tree was generated via TBtools software, in which bootstrap was set as 1,000.

### RNA isolation and RT-qPCR analysis

Total RNA was isolated from Arabidopsis seedlings using an RNAprep Pure Plant kit (Tiangen), after which it was reverse transcribed into cDNA using SuperScript III reverse transcriptase (Invitrogen). For RT-qPCR in Fig. 3, total RNA was extracted from 10 DAG seedlings. Quantitative assays were performed using a LightCycler 480 SYBR Green Master Mix (Roche). The internal control is ACTIN (Supplemental Table S3). Three biological replicates were performed in all experiments. Relative quantitative analysis was calculated as described previously (Zhou et al. 2022). The primers used for RT-qPCR are listed in Supplemental Table S3.

### DNA constructs and plant transformation

The genomic sequence of *MAC3A*, including the promoter and the *MAC3A* gene, was amplified using the primers MAC3ApromFLInF and MAC3ApromFLInR, and the genomic sequence of *MAC3B*, including the promoter and the *MAC3B* gene, was amplified using the primers MAC3BpromFLInF and MAC3BpromFLInR (Supplemental Table S3). The PCR products were cloned into the *pCAMBIA1300* vector with KpnI and HindIII sites using the Clontech In-Fusion HD Cloning kit to generate the *ProMAC3A:MAC3A-Flag* and *ProMAC3B:MAC3B-Flag* constructs. The plasmid *gMAC3A-Flag* was transformed into *mac3a-1 da3-1* plants, and the plasmid *gMAC3B-Flag* was transformed into *mac3b-1 da3-1* plants using *Agrobacterium tumefaciens* strain GV3101 via the floral dip method (Clough and Bent 1998). The transformants were then selected on a medium containing hygromycin (25 *μ*g/mL).

The *Pro35S:MAC3A-GFP* and *Pro35S:MAC3B-GFP* vectors were constructed using a PCR-based Gateway cloning system. The CDS of the *MAC3A* gene was amplified using the primers MAC3AGa-F and MAC3AGa-R, and the CDS of *MAC3B* was amplified using the primers MAC3BGa-F and MAC3BGa-R (Supplemental Table S3). The PCR products were subcloned into the *pDNOR-221* cloning vector (Invitrogen) with attB1 and attB2 sites, and *MAC3A* and *MAC3B* were cloned by the LR reaction into the binary vector *pGWB405* with attR1 and attR2 sites containing the CaMV35S promoter and the *GFP* gene. At the same time, *MAC3A* and *MAC3B* were cloned into the binary vector *pGWB417* with attR1 and attR2 sites containing the 35S promoter and the *Myc* CDS by the LR reaction to generate the *Pro35S:MAC3A-Myc* and *Pro35S:MAC3B-Myc* vector constructs. The 4 plasmids were transformed separately into WT plants using *Agrobacterium* GV3101. Transformants were selected on a medium containing kanamycin (25 *μ*g/mL).

### Subcellular protein localization

The *35S:GFP*, *35S:MAC3A-GFP*, *35S:MAC3B-GFP*, and *35S:UBP14-GFP* constructs were coexpressed transiently in Arabidopsis leaf protoplasts. The fluorescence signals in the protoplasts were observed by a laser confocal fluorescence microscope (LSM980, Carl Zeiss, Germany) using a 1% intensity excitation 488 nm laser with detection wavelength of 491 to 570 nm and detector gain of 799 V for GFP and a 0.8% intensity excitation 561 nm laser with detection wavelength of 588 to 632 nm and detector gain of 793 V for mCherry. mCherry-H2B was used as nuclear localization marker.

### BiFC assays

The CDSs of *MAC3A* and *MAC3B* were cloned into the vector *pSAT1-nVenus-C* (*pE3228*) to generate the plasmids *35S:MAC3A-nYFP* and *35S:MAC3B-nYFP*, and the CDS of *UBP14* was cloned into the vector *pSAT1-cCFP-C* (*pE3242*) to create the plasmid *35S:UBP14-cYFP*. Combinations of *MAC3A-nYFP*/*UBP14-cYFP*, *MAC3B-nYFP*/*UBP14-cYFP*, *MAC3A-nYFP*/*cYFP*, *MAC3B-nYFP*/*cYFP*, and *nYFP*/*UBP14-cYFP* were coinfiltrated into *Arabidopsis* protoplast cells and incubated for 12 to 16 h, after which the cells were observed using a laser scanning confocal microscope. YFP fluorescence signals were detected by a laser confocal fluorescence microscope (LSM980, Carl Zeiss, Germany) using a 1% intensity excitation 514 nm laser with detection wavelength of 526 to 570 nm and detector gain of 797 V for YFP.

### CoIP

The CDSs of *MAC3A* and *MAC3B* were cloned separately into the vector *pCsTMV1300-HA* to generate the plasmids *Pro35S:MAC3A-HA* and *Pro35S:MAC3B-HA*, and the CDSs of *UBP14* were cloned into the vector *pCsTMV1300-GFP* to generate the plasmids *Pro35S:UBP14-GFP*. Combinations of *MAC3A-HA* and *UBP14-GFP*, *MAC3A-HA* and *GFP*, *MAC3B-HA* and *UBP14-GFP*, *MAC3B-HA*, and *GFP* in *Agrobacterium* GV3101 were infiltrated into *N. benthamiana* leaves. Total proteins were extracted from leaf tissue in lysis buffer (50 mM HEPES pH 7.5, 100 mM NaCl, 10 mM EDTA pH 8.0, 0.2% [*v*/*v*] NP-40, 10% [*v*/*v*] glycerol, 2 mM DTT, and cOmplete Protease Inhibitor Cocktail [Roche]) and incubated with GFP-Trap-A agarose beads for 3 h at 4°C. The beads were then washed 4 times with wash buffer (50 mM HEPES pH 7.5, 250 mM NaCl, 10 mM EDTA pH 8.0, 0.1% [*v*/*v*] NP-40, 10% [*v*/*v*] glycerol). The immunoprecipitated fractions were separated by electrophoresis on 10% (*w*/*v*) SDS–PAGE and detected by immunoblot analysis with anti-GFP or anti-HA antibody.

### Pull-down assays

The CDSs of *MAC3A* and *MAC3B* were cloned separately into the vector *pET-28a* to generate the *His-MAC3A* and *His-MAC3B* constructs, respectively. The CDSs of *UBP14* were cloned into the vector *pGEX-4T-1* to generate the *GST-UBP14* construct. Constructs were transferred into *Escherichia coli* BL21 (DE3) cells. All proteins were expressed in *E. coli* BL21 (DE3) with 0.5 mM isopropyl β-D-1-thiogalactopyranoside (IPTG) at 28°C for 2 h. BL21 (DE3) cells were collected and resuspended with TGH buffer (50 mM HEPES pH 7.5, 150 mM NaCl, 1.5 mM MgCl_2_, 1 mM EGTA, 1% [*v*/*v*] Triton X-100, 10% [*v*/*v*] glycerol, 1 mM PMSF, and cOmplete Protease Inhibitor Cocktail [Roche]). Proteins were obtained from bacteria after sonicating for 5 min (5 s on, 15 s off) at 10 V. The combinations of GST, GST-UBP14, and His-MAC3A/B proteins were incubated with glutathione sepharose beads (GE Healthcare) at 4°C for 1 h. The beads were then washed 4 times with 1 mL TGH buffer. The precipitates were separated by electrophoresis on 10% (*w*/*v*) SDS–PAGE and detected by immunoblot analysis with anti-GST or anti-His antibody, respectively.

### Protein stability analysis

11-d-old WT, *mac3a-1*, *mac3b-1*, and *mac3a-1 mac3b-1* seedlings were incubated in liquid 1/2 MS medium containing 100 *μ*M CHX with/without 50 *μ*M MG132 for 3 h at 22°C. Total proteins were extracted with lysis buffer and subjected to SDS–PAGE and immunoblot analysis using anti-UBP14 (Xu et al. 2016) anti-Actin antibodies. The ImageJ software was used to measure the intensities of UBP14 bands and corresponding Actin bands on the blots. The UBP14 protein levels were shown as expression relative to Actin. The values of *mac3a-1*, *mac3b-1*, and *mac3a-1 mac3b-1* under CHX treatment and WT, *mac3a-1*, *mac3b-1*, and *mac3a-1 mac3b-1* under CHX + MG132 treatment were shown as the mean ± Sd (*n* = 3 biological replicates) relative to the value of WT under CHX treatment, set at 1.

To detect the protein levels of CDKB1;1, total proteins of 11-d-old WT, *mac3a-1*, *mac3b-1*, and *mac3a-1 mac3b-1* seedlings were extracted with lysis buffer. The immunoblot analysis was performed with anti-CDKB1;1 (PhytoAB, PHY0912S) and anti-Actin antibodies, respectively. The ImageJ software was used to measure the intensities of CDKB1;1 bands and corresponding Actin bands on the blots. The CDKB1;1 protein levels were shown as expression relative to Actin. The values in *mac3a-1*, *mac3b-1*, and *mac3a-1 mac3b-1* were shown as the mean ± Sd (*n* = 3 biological replicates) relative to the value of WT, and the value of WT is set at 1.

### In vivo ubiquitination assay

Protein A/G magnetic beads (B23202, Selleck) were equilibrated by lysis buffer. 10 *μ*L UBP14 antibody was incubated with 20 *μ*L protein A/G magnetic beads in the lysis buffer for 2 h at 4°C to combine into UBP14-Trap-A/G magnetic beads.

11-d-old *Arabidopsis* seedlings of WT, *mac3a-1* mutant, and *mac3a-1 mac3b-1* double mutant were incubated in liquid 0.5× MS medium containing 50 *μ*M MG132 for 3 h at 22°C. The total proteins of the seedlings were extracted with lysis buffer and incubated with UBP14-Trap-A/G magnetic beads overnight at 4°C. Beads were washed 3 times with wash buffer. The immunoprecipitated fractions were separated by electrophoresis on 8% (*w*/*v*) SDS–PAGE and detected by immunoblot analysis with anti-UBP14 or anti-Ub antibody.

### Total plant protein extraction and immunoblot assays

To detect the protein levels of UBP14, total proteins of 11-d-old *da3-1*, *mac3a-1 da3-1*, and *mac3b-1 da3-1* seedlings were extracted with lysis buffer (50 mM HEPES pH 7.5, 100 mM NaCl, 10 mM EDTA pH 8.0, 0.2% [*v*/*v*] NP-40, 10% [*v*/*v*] glycerol, 2 mM DTT, and cOmplete Protease Inhibitor Cocktail [Roche]). The immunoblot analysis was performed with anti-UBP14 and anti-Actin antibodies. The ImageJ software was used to measure the intensities of UBP14 bands and corresponding Actin bands on the blots. UBP14 protein levels were shown as expression relative to Actin. The values in *mac3a-1 da3-1* and *mac3b-1 da3-1* were shown as the mean ± Sd (*n* = 3 biological replicates) relative to the value of *da3-1*, and the value of *da3-1* is set at 1.

### Accession numbers

Sequence data from this article can be found in the GenBank/EMBL data libraries under the following accession numbers: *UBP14/DA3* (AT3G20630), *MAC3A/SUD1* (AT1G04510), *MAC3B/SUD2* (AT2G33340), and *CDKB1;1* (AT3G54180).

## Supplementary Material

kiad559_Supplementary_Data

## Data Availability

The data supporting this study's findings are available from the corresponding author upon reasonable request.

## Supplemental data

The following materials are available in the online version of this article.

**Supplemental Figure S1.** Identification of *da3-1* suppressors.

**Supplemental Figure S2.** Sequence alignment and phylogenetic analysis of MAC3A and MAC3B.

**Supplemental Figure S3.** The *mac3a-1* and *mac3b-1* mutations suppress the petal area of *da3-1*.

**Supplemental Figure S4.** The *mac3a-1 mac3b-1* double mutant suppresses the *da3-1* phenotype.

**Supplemental Figure S5.** Subcellular localization of MAC3A, MAC3B, and UBP14/DA3.

**Supplemental Figure S6.** The replicated assays are related to Fig. 5.

**Supplemental Figure S7.** The replicated assays are related to Fig. 5.

**Supplemental Figure S8.** The replicated assays are related to Fig. 6.

**Supplemental Table S1.** Identification of the *sud1-1* mutation using the MutMap approach.

**Supplemental Table S2.** Identification of the *sud2-1* mutation using the MutMap approach.

**Supplemental Table S3.** List of primers utilized in this study.

## Dive Curated Terms

The following phenotypic, genotypic, and functional terms are of significance to the work described in this paper:

UVI4 Gramene: AT2G42260
UVI4 Araport: AT2G42260

AT1G04510 Gramene: AT1G04510
AT1G04510 Araport: AT1G04510

AT2G33340 Gramene: AT2G33340
AT2G33340 Araport: AT2G33340

CHX Gramene: cycloheximide
CHX Araport: cycloheximide

DCL1 Gramene: AT1G01040
DCL1 Araport: AT1G01040

UBP14 Gramene: AT3G20630
UBP14 Araport: AT3G20630

CDKB1;1 Gramene: AT3G54180
CDKB1;1 Araport: AT3G54180
