# Supplementary material for: E3 ligases MAC3A and MAC3B ubiquitinate UBIQUITIN-SPECIFIC PROTEASE14 to regulate organ size in Arabidopsis
Source: Plant Physiol. 2023 Oct 18;194(2):684–97. doi: 10.1093/plphys/kiad559 (PMC10828200; doi:10.1093/plphys/kiad559)
Supplement: kiad559_Supplementary_Data [file kiad559_supplementary_data.zip › PP2023RA00938R1_Supplemental_Figures_1_8 and Tables_1_3.pdf]

Figure S1

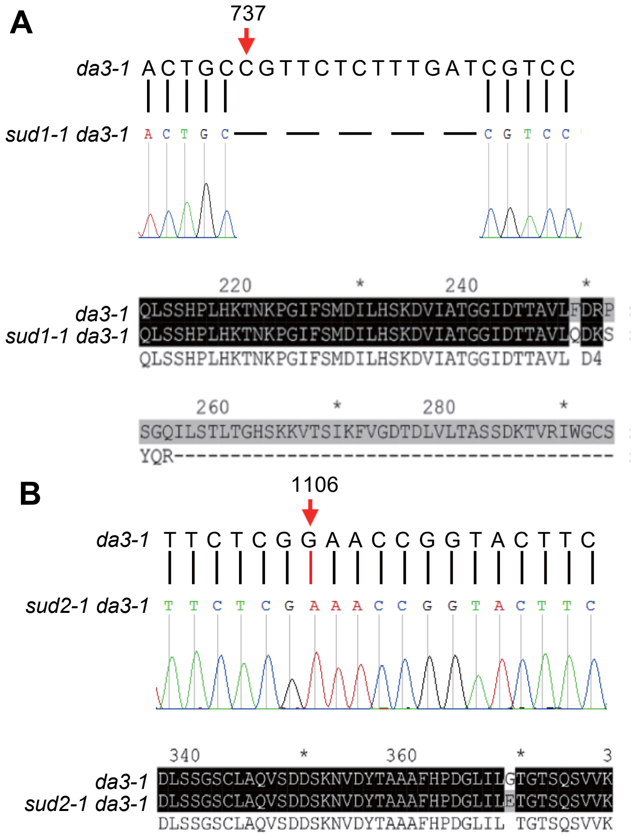

Supplemental Figure S1. Identification of *da3-1* suppressors.

(A) Sanger sequencing peak map (top panel) and amino acid sequence alignment (bottom panel) in *da3-1* and *sud1-1 da3-1*. The red arrow indicates that *SUD1-1* in *sud1-1 da3-1* has a 13 nucleotides deletion from the position 737 in the coding sequence, resulting in a premature stop codon.

(B) Sanger sequencing peak map (top panel) and amino acid sequence alignment (bottom panel) in *da3-1* and *sud2-1 da3-1*. The red arrow indicates that *SUD2* in *sud2-1 da3-1* has a G to A substitution at position 1106 in the coding sequence, resulting in an amino acid change from glycine to glutamic acid at position 369 of the protein.

Figure S2

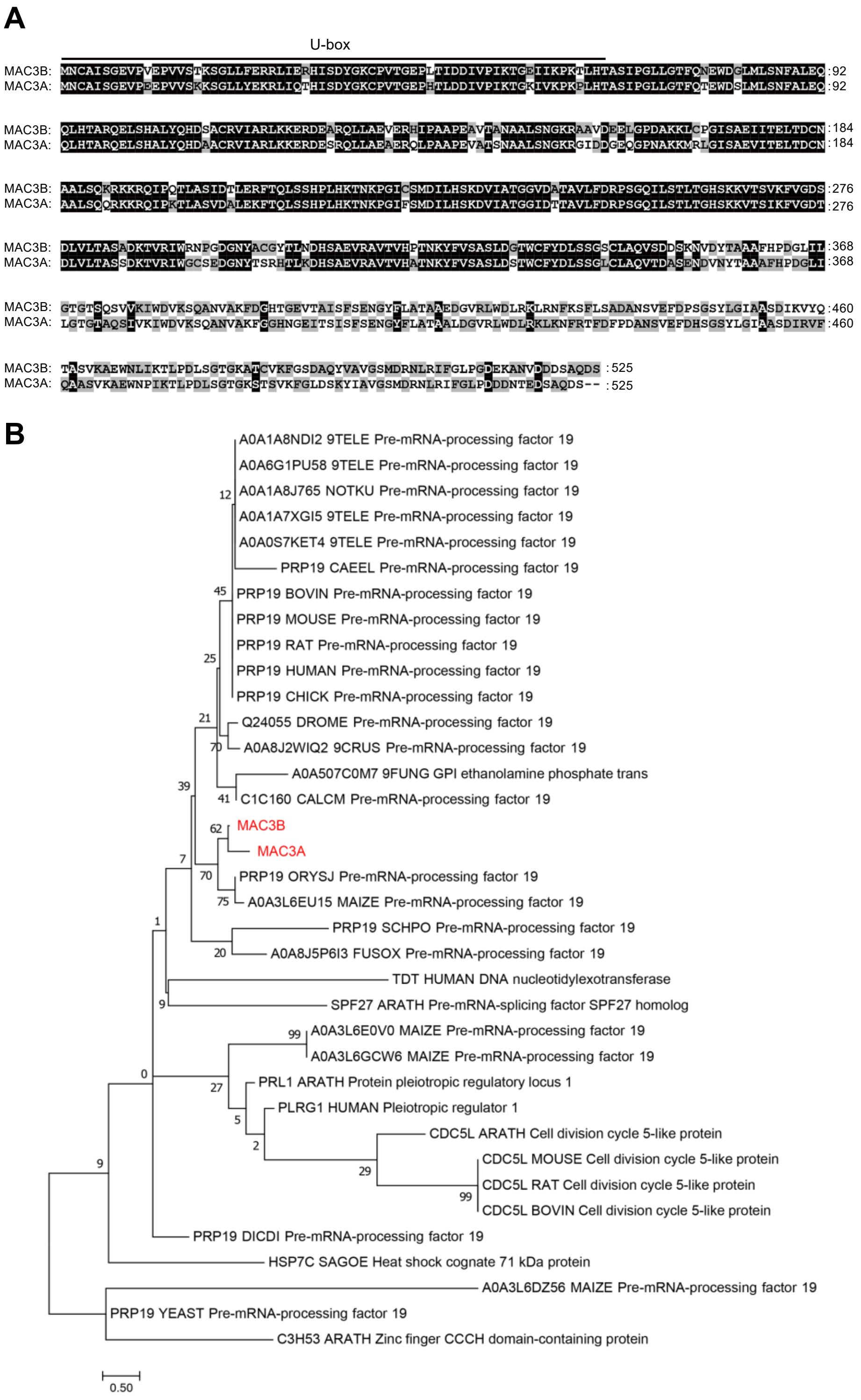

Supplemental Figure S2. Sequence alignment and phylogenetic analysis of MAC3A and MAC3B.

(A) Protein alignment of MAC3A and MAC3B. MAC3A and MAC3B protein sequences were downloaded from the NCBI database (https://www.ncbi.nlm.nih.gov/). The alignments were conducted using Genedoc software.

(B) Prp19 homologs from multiple species were selected from UniProt database (https://www.uniprot.org/), and Maximum likelihood tree was generated via MEGA7 software. The tree is drawn to scale, with branch lengths measured in the number of substitutions per site. The analysis involved 36 amino acid sequences. All positions containing gaps and missing data were eliminated.

Figure S3

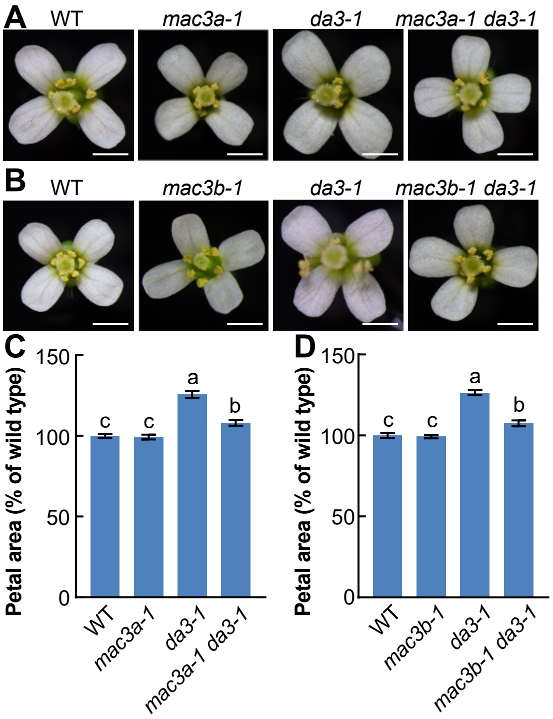

Supplemental Figure S3. The *mac3a-1* and *mac3b-1* mutations suppress the petal area of *da3-1*.

(A) Flowers of wild-type (WT, Col-0), *mac3a-1*, *da3-1*, and *mac3a-1 da3-1* (from left to right). Bars = 0.1 cm.

(B) Flowers of WT, *mac3b-1*, *da3-1*, and *mac3b-1 da3-1* (from left to right). Bars = 0.1 cm.

(C) Petal area of WT, *mac3a-1, da3-1*, and *mac3a-1 da3-1* (n = 60).

(D) Petal area of WT, *mac3b-1, da3-1*, and *mac3b-1 da3-1* (n = 60). Data represent mean values ± SE. Different lowercase letters indicate a significant difference among different groups, as determined by ANOVA (analysis of variance) and Tukey's post-hoc test ($P < 0.05$). Values in (C-D) are given as mean ± SE relative to the wild-type values, set at 100%.

Figure S4

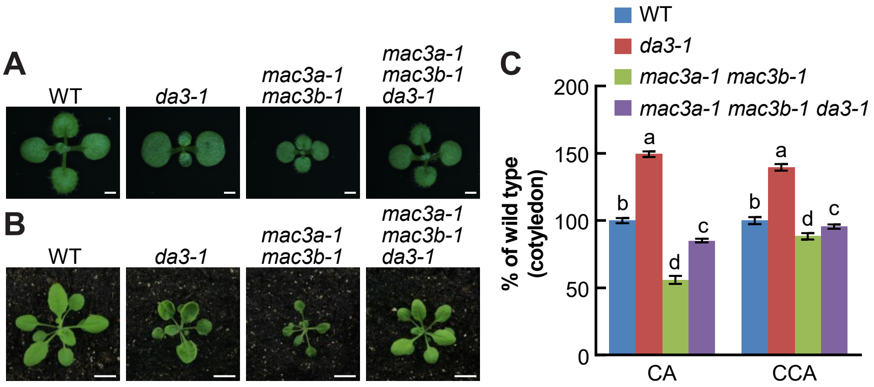

Supplemental Figure S4. The *mac3a-1 mac3b-1* double mutant suppresses the *da3-1* phenotype.

(A–B) The *mac3a-1 mac3b-1* double mutant suppresses organ growth phenotypes of *da3-1*. Eleven-day-old seedlings (A) and 24-day-old plants (B) of wild-type (WT, Col-0), *da3-1*, *mac3a-1 mac3b-1* and *mac3a-1 mac3b-1 da3-1* (from left to right). Bars = 0.1 cm in (A) and 1 cm in (B), respectively.

(C) Cotyledon area (CA) and cotyledon cell area (CCA) of 11-day-old WT, *da3-1*, *mac3a-1 mac3b-1* and *mac3a-1 mac3b-1 da3-1* seedlings (n = 40 for CA; n = 30 for CCA). Data are mean values ± SE. Different lowercase letters indicate a significant difference between diverse groups, determined by ANOVA (analysis of variance) and Tukey's post-hoc test ($P < 0.05$). Values are presented as mean ± SE relative to the wild-type values, set at 100%.

Figure S5

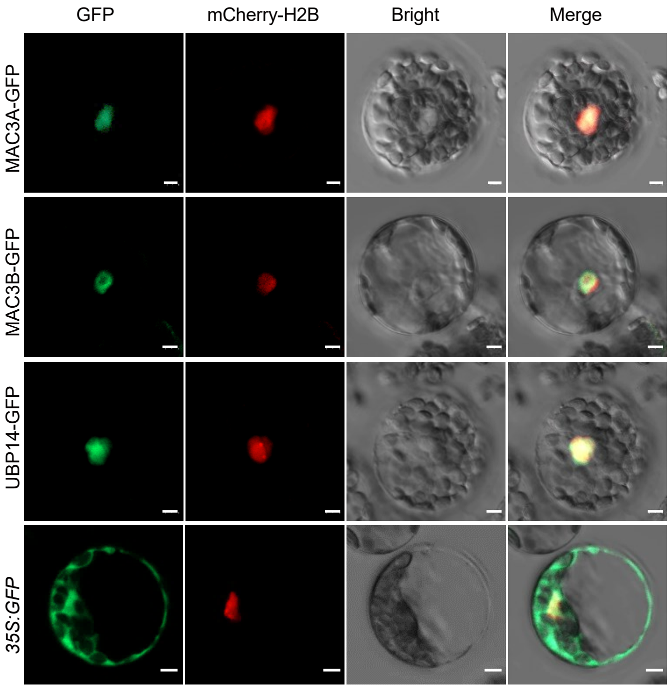

Supplemental Figure S5. Subcellular localization of MAC3A, MAC3B, and UBP14/DA3.

Subcellular localization of MAC3A-GFP (Green Fluorescent Protein), MAC3B-GFP, and UBP14-GFP in *Arabidopsis* protoplasts. *35S:GFP* was used as the control. Protoplasts were prepared from one-month-old soil-grown seedlings and transformed with 10 µg plasmid DNA (*35S:GFP*, *35S:MAC3A-GFP*, *35S:MAC3B-GFP*, and *35S:UBP14-GFP*). *35S:GFP* was utilized as a control. mCherry-H2B was used as nuclear localization marker. GFP and mCherry fluorescence were detected by a Zeiss LSM980 laser confocal microscope. Bars = 5 µm.

Figure S6

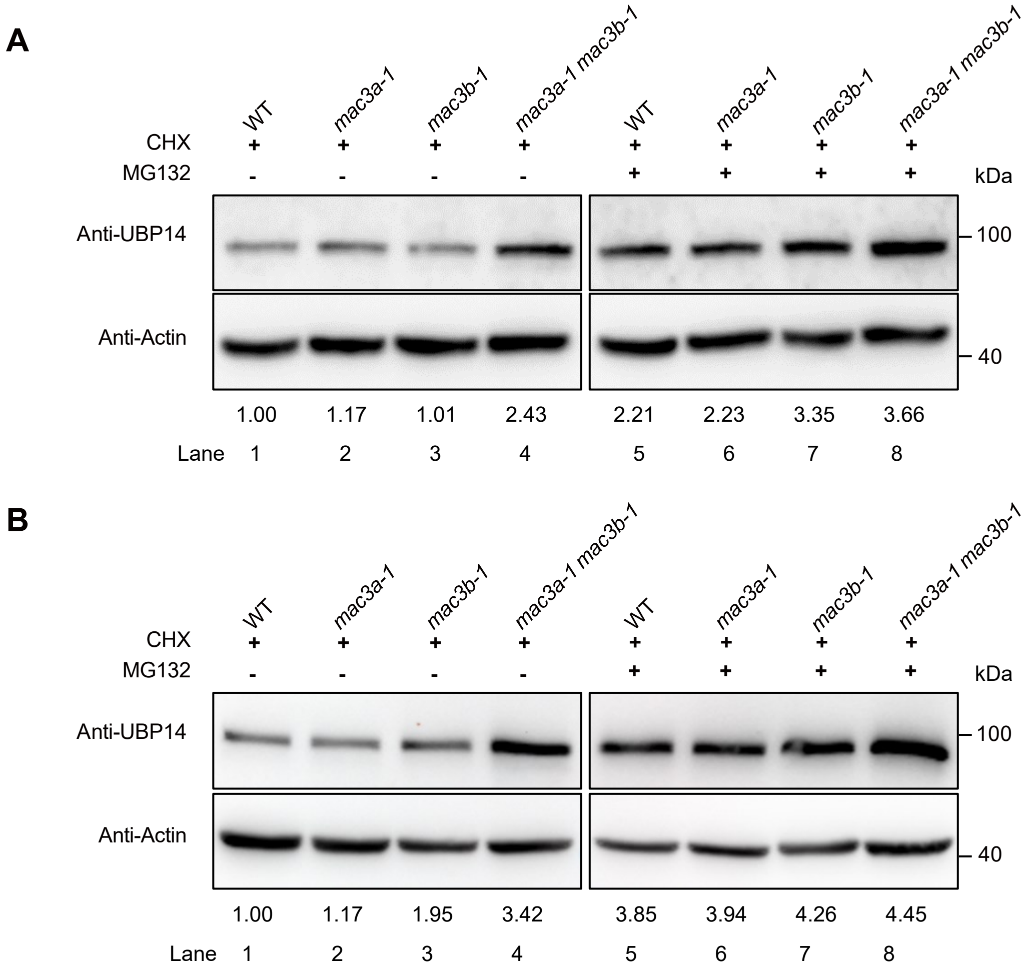

Supplemental Figure S6. The replicated assays are related to Figure 5.

MAC3A and MAC3B ubiquitinate UBP14 and regulate its stability. (A-B) UBP14 protein levels were detected by Western blot in wild-type (WT, Col-0), *mac3a-1*, *mac3b-1*, and *mac3a-1 mac3b-1* plants. 11-day-old WT, *mac3a-1*, *mac3b-1*, and *mac3a-1 mac3b-1* seedlings were pretreated using 100 µM cycloheximide (CHX) in the absence or presence of 50 µM MG132 for 3 hours. Whole seedlings were harvested, and total protein was extracted. An anti-UBP14 specific antibody was used to detect UBP14. Actin was used as a loading control. Relative protein levels of UBP14 were calculated relative to the value of WT under CHX treatment (artificially set at 1.0).

Figure S7

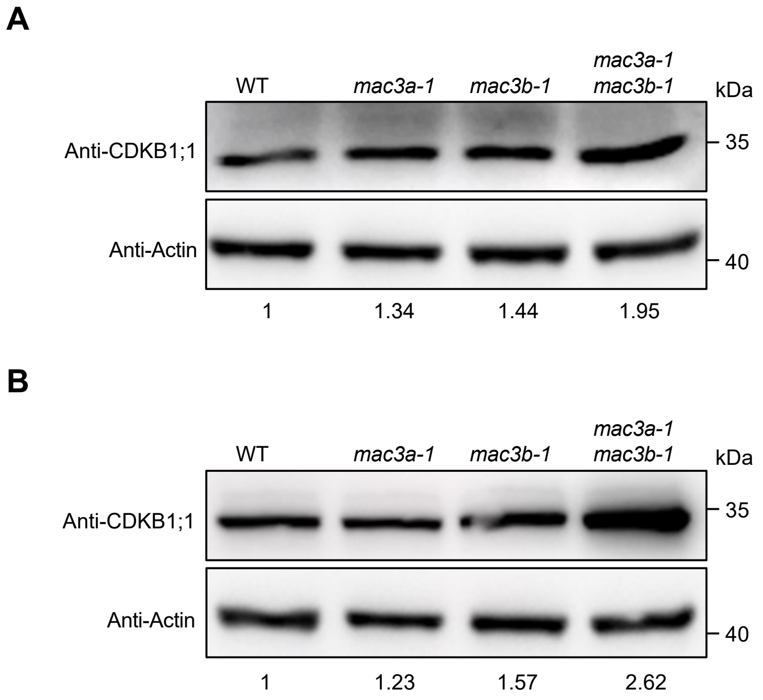

Supplemental Figure S7. The replicated assays are related to Figure 5.

MAC3A and MAC3B modulate the stability of CDKB1;1. (A-B) CDKB1;1 protein level was detected by Western blotting in WT, *mac3a-1*, *mac3b-1*, and *mac3a-1 mac3b-1* plants. 11-day-old seedlings were harvested, and total protein was extracted. Anti-CDKB1;1 specific antibody was employed to detect protein levels. Actin was used as a loading control. CDKB1;1 relative protein level was calculated relative to Actin. Relative protein levels of CDKB1;1 was calculated relative to the value of WT (artificially set at 1.0).

Figure S8

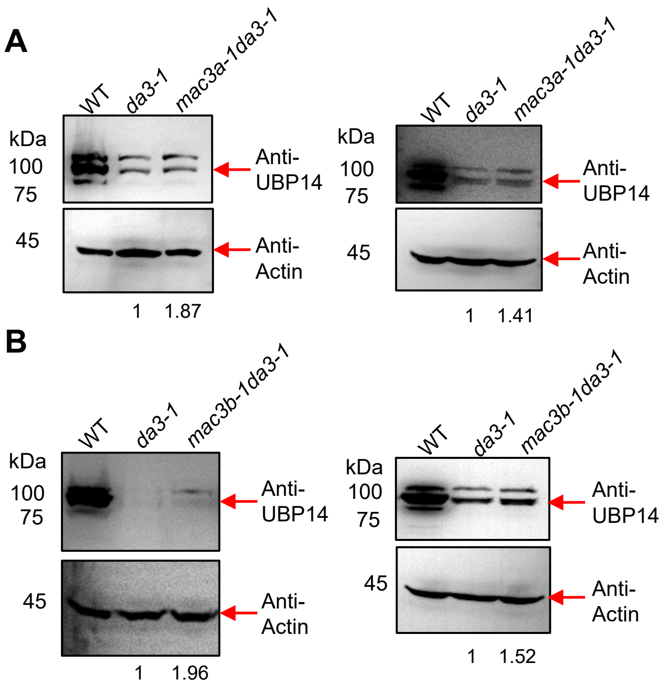

Supplemental Figure S8. The replicated assays are related to Figure 6.

mac3a-1 and *mac3b-1* mutants rescued the UBP14 protein level of *da3-1* (UBP14 partial loss function mutant). (A-B) UBP14 protein abundance was evaluated by Western blotting in 11 DAG wild-type (WT, Col-0), *da3-1*, and either *mac3a-1 da3-1* (A) or *mac3b-1 da3-1* (B) seedlings. Total proteins were extracted from seedlings and separated by electrophoresis. Anti-UBP14-specific antibodies were used to detect UBP14 in Western blots. Actin was used as a loading control to compute the relative abundance of UBP14.

No.	chromosome	position	*da3-1*	*sud1-1 da3-1*	*da3-1*.GT	*da3-1*.AD	*sud1-1 da3-1*.GT	*sud1-1 da3-1*.AD	Gene	Note	SNP/INDEL index
INDEL71	1	1228740	CCGTTCTCTTTGAT	C	CCGTTCTCTTTGAT/CCGTTCTCTTTGAT	27,0	C/C	0,22	AT1G04510	Exonic, deletion	1
SNP591	1	6949585	G	A	G/G	37,0	A/A	0,5	AT1G20050	Intronic	1
SNP765	1	9103828	C	T	C/C	17,0	T/T	0,16	AT1G26310	upstream	1

Supplemental Table S1. Identification of the *sud1-1* mutation using the MutMap approach.

The whole genome sequencing and mutated loci analysis reveal there are three candidate genes associated with the *sud1-1* mutation. GT: genotype; AD: Allele Depth. "Intronic" or "Exonic" represents the mutation happens in intronics or exons of the indicated gene.

No.	chromo some	position	*da3-1*	*sud2-1 da3-1*	*da3-1*.GT	*da3-1*.AD	*sud2-1 da3-1*.GT	*sud2-1 da3-1*.AD	Gene	Note	SNP/INDEL index
SNP10790	2	6421840	G	A	G/G	23,0	A/A	0,7	AT2G14930	downstream	1
SNP11636	2	13729964	C	T	C/C	30,0	T/T	0,22	AT2G32320	Intronic	1
SNP11677	2	14127512	C	T	C/C	25,0	T/T	0,23	AT2G33340	Exonic	1

Supplemental Table S2. Identification of the *sud2-1* mutation using the MutMap approach.

The whole genome sequencing and mutated loci analysis reveal there are three candidate genes associated with the *sud2-1* mutation. GT: genotype; AD: Allele Depth. “Intronic” or “Exonic” represents the mutation happens in intronics or exons of the indicated gene.

Supplemental Table S3. List of primers utilized in this study.

Primer Name	DNA sequence (5'-3')
**Primers for T-DNA identification**	
mac3a-1-LP	CGATGCTCTGGAGAAGTTCAC
mac3a-1-RP	ATTTTGGAGACCTGAGCAAGG
mac3b-1-LP	TTTGATAGCATCAAACCGTCC
mac3b-1-RP	CCGGAGAAGATAAAACCCTTG
LBb1.3	ATTTTGCCGATTTCGGAAC
da3-1-Mbo1-F	GAGAAAGCAACAGACTGGGTATTCAACAAC
da3-1-Mbo1-R	GACTCAAAAGAGGGTTTAAGAAACTAG
**Primers for QRT-PCR**	
MAC3A-qRT-F1	GCTGACTGGTCACTCAAAGAA
MAC3A-qRT-R1	GTTCCCATCCTCGGAACATC
MAC3B-qRT-F2	ACGAAGTCAGGATTACTCTTCG
MAC3B-qRT-R2	GTCATCAATGGTAAGTGGTTCG
ACTIN-qRT-F	GTAACATTGTGCTCAGTGGTGGTA
ACTIN-qRT-R	GATAGAACCACCAATCCAGACACT
**Primers for constructs**	
MAC3ApromFLInF	GGTACCCGGGGATCCTCGGTAAAGGAGTTTGACCCAAT
MAC3ApromFLInR	AAGCTTGCATGCCTGCATCAGATCTTATCGTCGTCATCCTTGTAATCTGAATCTTGTGCTGAATCTTCA
MAC3BpromFLInF	GGTACCCGGGGATCCTGTTTGAGCATAGGGTCAATGCA
MAC3BpromFLInR	AAGCTTGCATGCCTGCATCAGATCTTATCGTCGTCATCCTTGTAATCCGAGTCTTGCGCAGAGTCATCA
MAC3AGa-F	GGGGACAAGTTTGTACAAAAAAGCAGGCTGCATGAATTGTGCAATTTCCGGCG
MAC3AGa-R	GGGGACCACTTTGTACAAGAAAGCTGGGTGTGAATCTTGTGCTGAATCTTCAGT
MAC3BGa-F	GGGGACAAGTTTGTACAAAAAAGCAGGCTGCATGAACTGTGCAATTTCAGGAG
MAC3BGa-R	GGGGACCACTTTGTACAAGAAAGCTGGGTGCGAGTCTTGCGCAGAGTCATC
**Primers for BiFC**	
nYFPMAC3A-F	CGCCACAACATCGAGAGATCTATGAATTGTGCAATTTCCGGC
nYFPMAC3A-R	GCTTCGAACTCGAGCTCTAGATCATGAATCTTGTGCTGAATCTTCA
nYFPMAC3B-F	CGCCACAACATCGAGAGATCTATGAACTGTGCAATTTCAGGAGAA
nYFPMAC3B-R	GCTTCGAACTCGAGCTCTAGACGAGTCTTGCGCAGAGTCATC
cYFPDA3-F	TACAAGTCCGGACTCAGATCTATGGAGCTCCTCCGATCCAA
cYFPDA3-R	CCGCGGTACCGTCGACTGCAGTCAATCAAGCCGCTGAAAGAA
**Primers for CoIP**	
MAC3AHAInF	GGTACCGCGGGCCCGGGATCCATGAATTGTGCAATTTCCGGC
MAC3AHAInR	TGGGTAACCTGCCATGGATCCCATGAATCTTGTGCTGAATCTTCAG
MAC3BHAInF	GGTACCGCGGGCCCGGGATCCATGAACTGTGCAATTTCAGGAGAA
MAC3BHAInR	TGGGTAACCTGCCATGGATCCCACGAGTCTTGCGCAGAGTCA
DA3InGFPF	GGTACCGCGGGCCCGGGATCCATGGAGCTCCTCCGATCCAA
DA3InGFPR	CCTTTACTCATCCATGGATCCCAATCAAGCCGCTGAAAGAAG
**Primers for Pull-down**	
His-MAC3AF	CGGGATCCATGAATTGTGCAATTTCCGGCG
His-MAC3AR	ACGCGTCGACTCATGAATCTTGTGCTGAATCTTC
His-MAC3BF	CGGGATCCATGAACTGTGCAATTTCAGGAG
His-MAC3BR	ACGCGTCGACTCACGAGTCTTGCGCAGAGTC
GST-DA3F	CGGGATCCATGGAGCTCCTCCGATCCAAC
GST-DA3R	ACGCGTCGACTCAATCAAGCCGCTGAAAGAAGTA
