# Supplementary material for: E3 ligases MAC3A and MAC3B ubiquitinate UBIQUITIN-SPECIFIC PROTEASE14 to regulate organ size in Arabidopsis
Source: Plant Physiol. 2023 Oct 18;194(2):684–97. doi: 10.1093/plphys/kiad559 (PMC10828200; doi:10.1093/plphys/kiad559)
Supplement: kiad559_Supplementary_Data [file PP2023RA00938R1_Merged_Decision_Summary_PDF.pdf › PP2023RA00938R1_Merged_Decision_Summary_PDF.pdf]

Plant Physiology®

MAC3A and MAC3B ubiquitinate UBP14 to regulate organ size in Arabidopsis thaliana

Xiaopeng Guo, Xin Zhang, Shan Jiang, Xin Qiao, Bolun Meng, Xiaohang Wang, Yanan Wang, Kaihuan Yang, Yilan Zhang, Na Li, Tianyan Chen, Yiyang Kang, Mengyi Yao, Xuan Zhang, Xinru Wang, Erling Zhang, Junhua Li, Dawei Yan, Zhubing Hu, Jose Botella, Chun-Peng Song, Yunhai Li, and Siyi Guo

Review Timeline:

Submission Date:	22-Jun-2023
Editorial Decision:	30-Jul-2023
Revision Received:	12-Sep-2023
Accepted:	22-Sep-2023

30-Jul-2023

Prof. Dr. Siyi Guo
State Key Laboratory of Crop Stress Adaptation and Improvement, Henan University
Kaifeng, Henan
China

RE: MAC3A and MAC3B ubiquitinate UBP14 to regulate organ size in Arabidopsis thaliana

Dear Dr. Guo:

We have received reviews of your manuscript, which you may view at the end of this letter. On the basis of the advice received and my own evaluation, I believe that, pending revision, this article may ultimately be acceptable for publication in Plant Physiology.

Both reviewers agreed that the data in your manuscript expands our understanding of molecular mechanism underlying organ development and organ size control in Arabidopsis. However, they also raise some questions and suggestions for you to improve the quality of the manuscript. Please reply one by one when providing the revised manuscript.

When you are ready to submit your revision, click the below link and find the red arrow under your Author Tasks heading for this revision submission.

If you want to visit this version of the manuscript, you may click the link below and then click on the folder that reads "Post Decision Manuscripts".

Link Not Available

When uploading the revised version of this article, please be sure to include as complete an answer as possible to the referees' criticisms during the first round of reviews in the "response to reviewers" section.

Plant Physiology considers a revision for up to 60 days after a manuscript has been provisionally accepted; limited extension of this timeframe is at the discretion of the Monitoring Editor. A reminder notice will be sent to you 2 weeks before the deadline, at which time you may choose to contact the editorial office to request an extension; if so, please provide a justification for your request. After the expiration of this deadline, the article would need to be submitted as a resubmission and would be handled as such.

Thank you very much for giving us an opportunity to review this work. I look forward to receiving the next version.

Sincerely,

Dr. Haiyun Ren
Monitoring Editor, Plant Physiology

Want to add this revision deadline to your calendar? Click below!

-- Reviewer comments:
Reviewer #1 (Comments for the Author):

The organ size is critical for plant development and especially crop yields, and many factors and pathways have been identified to influence organ development and thus organ size. In this manuscript, authors identified two suppressors of the Arabidopsis da3-1, in which the mutation in two respective E3 ligase genes MAC3A and MAC3B partially suppresses the high ploidy and organ size phenotypes of the da3-1. Biochemical and genetic evidences further demonstrate that MAC3A and MAC3B physically interact with and ubiquitinate UBP14/DA3 to destabilize its stability, and thus leading the degradation of CDKB1;1 to control cell endoreduplication and organ size. This work defines MAC3A and MAC3B as the regulatory components of UBP14-CDKB1;1-

mediated endoreduplication and thus organ size in Arabidopsis.
Overall, the work expands our understanding of molecular mechanism underlying organ development and organ size control. The genetic and biochemical evidences are substantive for supporting the conclusions. However, a few concerns regarding the organization and writing are necessary to be addressed to improve the manuscript.

Specific points:

1. In first paragraph of introduction, authors need to give a brief introduction of how plant organ size is determined at a cellular level, including cell proliferation and expansion, especially the role of endoreduplication during cell size determination.
2. In lines 98-105, introduction of MAC3A and MAC3B did not give much information but largely affect the logic here. This should be combined into the results and/or discussion part.
3. Since CDKB1;1 has been shown to be downstream target of UBP14, it is predicable that MAC3A and MAC3B results in its degradation by UBP14. It is ambiguous to state that MAC3A and MAC3B promotes its degradation. This point should be clearly clarified at Abstract and text.
4. Col-0 is used the as control throughout the text, it is better to use WT instead.
5. It is more interesting to examine whether MAC3A and MAC3B are responsive to growth and environmental cues, which would provide information about the biological role of this regulatory module during organ development under ever-changed environmental response.
6. The writing should be improved for the manuscript, it is better to make an editing work on it.

Reviewer #2 (Comments for the Author):

Through a forward genetic screening, the authors identified the E3 ligases coding genes, MAC3A and MAC3B, function as suppressors of the da3-1 mutant and partially suppressed its phenotype in ploidy and organ size. The authors further found that MAC3A and MAC3B physically interact with UBP14/DA3 and regulate its stability through the ubiquitin proteasome pathway. The paper itself is a further in-depth study for the known UBP14/DA3-CDKB1; 1 module, and provides new information for us about the molecular mechanism underlying organ size control in Arabidopsis.
However there are some questions to address:
Major:
-- The result in Figure 6 was only mentioned in the discussion part (Line 311), but is not described in the results section. As this is an important data supporting the main points, it should be described in detail.
--Line 148-151. The process of gene cloning is written too brief, and I did not get any information about this in the Methods section. How did the authors construct the population for mapping? What's the separation ratio? How many were individual plants selected for sequencing? What's the reference genome? And so on.
--Line 146-147. "the sud1-1 and sud2-1 mutations partially suppress the high ploidy, cell and organ growth phenotypes observed in da3-1 mutants." Are there some other possible contributions for suppressing the phenotype of da3-1 mutants? A possible explanation? Please add this in the Discussion section.
--Line 261-262. "but the increase in UBP14/DA3 protein levels in Col-0 compared with the mac3a-1 mac3b-1 mutant was partially blocked". The meaning of the sentence is somewhat unclear. Please describe it in another way.
--Figure 5B. The picture showed for UBP14 ubiquitination analysis is blurry and the signal ladder of anti-ubiquitin is not clear. Are there any clearer pictures or results? In addition, quantitative statistics of ubiquitination levels of UBP14 should be added.

Minor:
-- Some sentences are not smooth, for example Line 144-145, Line 163, Line 207-208.
-- Please organize the pictures in an order according to the text. For example Line 187 Figure 3D, E.

Dear Reviewers,

We are very grateful for the reviewers' supportive and helpful comments on this manuscript (PP2023-RA-00938). We have carefully considered your suggestions in this revision and addressed the concerns raised by the reviewers. They have helped us craft an improved paper. Additional experiments were performed according to requirements, and we developed our point-by-point responses below. Please contact us if you have any questions or need any additional information. Thank you kindly for your help in this matter.

Yours sincerely,

Siyi Guo, Corresponding Author

guosiyi@henu.edu.cn

Tel: 86-371-23881387

Fax: 86-371-23881387

Reviewer #1 (Comments for the Author):

The organ size is critical for plant development and especially crop yields, and many factors and pathways have been identified to influence organ development and thus organ size. In this manuscript, authors identified two suppressors of the Arabidopsis da3-1, in which the mutation in two respective E3 ligase genes MAC3A and MAC3B partially suppresses the high ploidy and organ size phenotypes of the da3-1. Biochemical and genetic evidences further demonstrate that MAC3A and MAC3B physically interact with and ubiquitinate UBP14/DA3 to destabilize its stability, and thus leading the degradation of CDKB1;1 to control cell endoreduplication and organ size. This work defines MAC3A and MAC3B as the regulatory components of UBP14-CDKB1;1-mediated endoreduplication and thus organ size in Arabidopsis.
Overall, the work expands our understanding of molecular mechanism underlying organ development and organ size control. The genetic and biochemical evidences are substantive for supporting the conclusions.

However, a few concerns regarding the organization and writing are necessary to be addressed to improve the manuscript.

Specific points:

1. In first paragraph of introduction, authors need to give a brief introduction of how plant organ size is determined at a cellular level, including cell proliferation and expansion, especially the role of endoreduplication during cell size determination.

Response: Thank you for these suggestions. We have added the description of how plant organ size is determined by cell proliferation and expansion, and the functions of endoreduplication in cell size control (Line 68-73), as follows:

In plants, high ploidy levels are usually associated with cell and organ growth (Gegas et al., 2014; Jiang et al., 2022a; Jiang et al., 2022b; Xu et al., 2016). During organ enlargement, the endoreduplication mainly occurs in the cell differentiation process, that cells replicate their nuclear DNA without subsequently dividing, resulting in cells with higher ploidy levels (Breuer et al., 2014; Edgar et al., 2014; Lang and Schnittger, 2020).

2. In lines 98-105, introduction of MAC3A and MAC3B did not give much information but largely affect the logic here. This should be combined into the results and/or discussion part.

Response: Thanks for your suggestions. We have moved those sentences to the result part in the revision (Line 157-163).

3. Since CDKB1;1 has been shown to be downstream target of UBP14, it is predicable that MAC3A and MAC3B results in its degradation by UBP14. It is ambiguous to state that MAC3A and MAC3B promotes its degradation. This point should be clearly clarified at Abstract and text.

Response: Thank you for these suggestions. We have introduced this point in the Abstract (Line 52-55) as follows:

We have previously reported that, UBP14/DA3 acts upstream of the B-type cyclin-dependent kinase (CDKB1;1) and maintains its stability for inhibiting endoreduplication and cell growth. In this work, MAC3A and MAC3B were found to promote the degradation of CDKB1;1 by ubiquitinating UBP14/DA3.

This point is also included in the text (Line 277-284) as follows:

it is understandable that MAC3A and MAC3B impact the degradation through ubiquitination of UBP14. In this study, western blot analysis of total protein extracts from WT, *mac3a-1*, *mac3b-1*, and *mac3a-1 mac3b-1* plants was conducted. The results indicated that CDKB1;1 protein levels were increased in *mac3a-1 mac3b-1* mutants compared to WT (Figure 5C; Supplemental Figure S7). Taken together, MAC3A and MAC3B were found to promote the degradation of CDKB1;1 through ubiquitination of UBP14/DA3, resulting in a high level of endopolyploidy linked to organ enlargement.

4. Col-0 is used the as control throughout the text, it is better to use WT instead.

Response: Thanks for your suggestions. We have used “WT” instead of “Col-0” throughout the text.

5. It is more interesting to examine whether MAC3A and MAC3B are responsive to

growth and environmental cues, which would provide information about the biological role of this regulatory module during organ development under ever-changed environmental response.

Response: Thank you for these comments. We have detected the mRNA expression of MAC3A, MAC3B, and UBP14/DA3 (FigR1), during organ development under dynamic environmental response (e.g. GA treatment, NaCl treatment, cold treatment, heat treatment and drought treatment). In this study, we hope to focus on examining whether MAC3A and MAC3B are responsive to growth and environmental cues. Moreover, we have included previously reported results about the roles of MAC3 and MAC3B in environmental responses (Line 157-163).

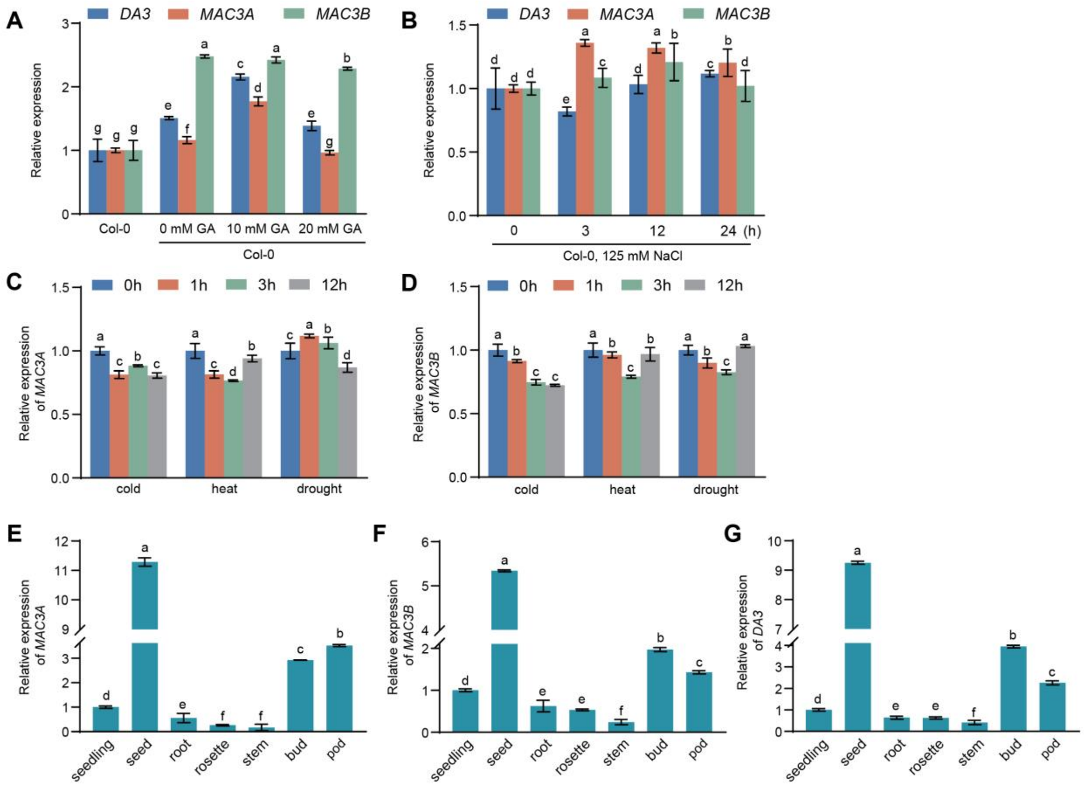

FigR1 mRNA expression of *MAC3A*, *MAC3B*, and *UBP14/DA3*, during organ development under dynamic environmental response.

6. The writing should be improved for the manuscript, it is better to make an editing work on it.

Response: Thank you for your suggestions. The writing of this manuscript has been improved by a native English editing expert.

Reviewer #2 (Comments for the Author):

Through a forward genetic screening, the authors identified the E3 ligases coding genes, MAC3A and MAC3B, function as suppressors of the da3-1 mutant and partially suppressed its phenotype in ploidy and organ size. The authors further found that MAC3A and MAC3B physically interact with UBP14/DA3 and regulate its stability through the ubiquitin proteasome pathway. The paper itself is a further

in-depth study for the known UBP14/DA3-CDKB1; 1 module, and provides new information for us about the molecular mechanism underlying organ size control in Arabidopsis.

However there are some questions to address:

Major:

1. The result in Figure 6 was only mentioned in the discussion part (Line 311), but is not described in the results section. As this is an important data supporting the main points, it should be described in detail.

Response: Thank you very much for your suggestions. We have emphasized this point in the corresponding portion of the results section (Line 285-299), as follows:

We have previously reported that the *da3-1* mutation results in a partial loss of function mutant of UBP14, and the UBP14$^{da3\text{-}1}$ mutation disrupts the deubiquitination activity of UBP14 (Xu et al., 2016). In the *da3-1* mutant, a low level of UBP14 protein was still detected (Figure 6A). We observed that *mac3a-1* and *mac3b-1* partially suppress the phenotype of *da3-1* by controlling endoreduplication. Moreover, our results demonstrate that MAC3A/3B ubiquitinates UBP14 *in vivo* and influences DA3 protein abundance. We hypothesized that the decreased ubiquitination levels of UBP14 in *mac3a-1* and *mac3b-1* caused the observed protein abundances of UBP14 in *mac3a-1 da3-1* and *mac3b-1 da3-1* double mutants. Therefore, *mac3a-1* and *mac3b-1* can suppress endoreduplication phenotypes of *da3-1*. Consistently, we identified the UBP14 protein levels in *mac3a-1 da3-1* and *mac3b-1 da3-1* double mutants, and both the mutations in MAC3A and MAC3B can partially rescue UBP14 protein levels in *da3-1* mutants (Figure 6, A-D; Supplemental Figure S8). These results support that *mac3a-1* and *mac3b-1* partially block the *da3-1* phenotype by partially enhancing DA3 protein abundance.

2. Line 148-151. The process of gene cloning is written too brief, and I did not get any information about this in the Methods section. How did the authors construct the population for mapping? What's the separation ratio? How many were individual plants selected for sequencing? What's the reference genome? And so on.

Response: Thank you for this suggestion. We have re-organized this section, and described it in detail as suggested. We have added the description in the “Materials and Methods” as well as Supplemental Table S1/S2, as follows:

The MutMap approach was conducted to identify the *sud1-1* mutations using the F2 population of a cross between *sud1-1 da3-1* and *da3-1*. In the F2 population, the separation ratio was 3:1, suggesting that a single recessive mutation determined the phenotypes of *sud1-1*. We extracted DNA from 72 plants in the F2 population that exhibited the *sud1-1 da3-1* phenotypes and mixed equal amounts of DNA from these plants for whole-genome sequencing. DNA from *da3-1* was sequenced as a control. We detected 33,036 SNPs (single nucleotide polymorphisms) and 7,267 INDELs (insertions and deletions) between the pooled F2 samples and *da3-1*. Given that all mutant plants in the F2 population were presumed to possess the causative SNP/INDEL, the SNP/ INDEL ratio for this causative mutation in bulk F2 plants

should be 1. In total, one INDEL and two SNPs had an SNP/INDEL-index = 1 (Supplemental Table S1). Only INDEL71 was identified in exons (Supplemental Table S1). We further developed the CAPS (Cleaved Amplified Polymorphic Sequences) marker based on the INDEL71 mutation, and found INDEL71 was co-segregated with the *sud1-1 da3-1* phenotypes. The INDEL71 contained a 13-bp deletion in *sud1-1* in the gene *AT1G04510*, resulting in a premature stop codon. These results suggested that *AT1G04510* was the candidate gene for *SUD1*.

The *sud2-1* mutation was identified utilizing a similar method to that of *sud1-1*. In total, three SNPs had an SNP/INDEL-index = 1 (Supplemental Table S2). Among these, only SNP11677 occurred in exons. SNP11677 involved a G to A substitution in the *AT2G33340* gene, causing a glycine to glutamic acid change. We developed a dCAPS marker based on this mutation and determined that SNP11677 co-segregated with the *sud2-1 da3-1* phenotypes. These results suggest that *AT2G33340* is the candidate gene of *SUD2*.

3. Line 146-147. "the *sud1-1* and *sud2-1* mutations partially suppress the high ploidy, cell and organ growth phenotypes observed in *da3-1* mutants." Are there some other possible contributions for suppressing the phenotype of *da3-1* mutants? A possible explanation? Please add this in the Discussion section.

Response: Thank you for your suggestions. We have discussed other possible contributions of MAC3A and MAC3B for suppressing the phenotype of *da3-1* mutants in the discussion section as recommended. Our results demonstrate that the DA3 protein abundances *mac3a-1 da3-1* and *mac3b-1 da3-1* double mutants are increased but lower than that in wild-type (Figure 6, A-D). Therefore, we hypothesized the *sud1-1* and *sud2-1* mutations increase DA3 protein levels but not to WT levels, thereby partially suppressing phenotypes of the *da3-1* mutant. MAC3A and MAC3B share high sequence homology and similarity and have ubiquitin ligase activity (Monaghan et al., 2009). MAC3A and MAC3B function redundantly in microRNA (miRNA) biogenesis, RNA splicing, and the regulation of immune responses in plants (Jia et al., 2017; Li et al., 2018), which suggests that there may also be functional redundancy in the regulation of nuclear replication and organ size. Moreover, the *sud1-1* and *sud2-1* mutations partially suppress the phenotypes of *da3-1* mutants, indicating there are other components required for the regulation of UBP14 to control endoreduplication. *In vivo* ubiquitination experiments indicated that some ubiquitination modifications of UBP14 were detected in *mac3a-1 mac3b-1* double mutant (Figure 5C), which may imply the presence of other factors working together to regulate the endoreduplication and organ size of *Arabidopsis*. Overall, the *sud1-1* and *sud2-1* mutations partially inhibit the high ploidy, cell, and organ growth phenotypes observed in *da3-1* mutants.

The part of "Our results demonstrate that the DA3 protein abundances *mac3a-1 da3-1* and *mac3b-1 da3-1* double mutants are increased but lower than that in wild-type (Figure 6, A-D). Therefore, we hypothesized the *sud1-1* and *sud2-1* mutations increase DA3 protein levels but not to WT levels, thereby partially suppressing phenotypes of the *da3-1* mutant." was integrated in the discussion (Line

339-343).

The part of "*In vivo* ubiquitination experiments indicated that some ubiquitination modifications of UBP14 were detected in *mac3a-1 mac3b-1* double mutant (Figure 5C), which may imply the presence of other factors working together to regulate the endoreduplication and organ size of *Arabidopsis*." was also integrated in the discussion (Line 346-348).

4. Line 261-262. "but the increase in UBP14/DA3 protein levels in Col-0 compared with the *mac3a-1 mac3b-1* mutant was partially blocked". The meaning of the sentence is somewhat unclear. Please describe it in another way.

Response: Thank you for your suggestions. We have re-organized this sentence and included it in the results section (Line 263-268), as follows:

The seedlings of WT, *mac3a-1*, *mac3b-1*, and *mac3a-1 mac3b-1* mutants were treated with MG132 (a proteasome inhibitor), and it showed that the destabilization of UBP14/DA3 mediated by both MAC3A and MAC3B was reversed by MG132 to some extent, suggesting that MAC3A and MAC3B downregulated the UBP14/DA3 protein through the ubiquitin-proteasome pathway (Figure 5A, right panel).

5. Figure 5B. The picture showed for UBP14 ubiquitination analysis is blurry and the signal ladder of anti-ubiquitin is not clear. Are there any clearer pictures or results? In addition, quantitative statistics of ubiquitination levels of UBP14 should be added.

Response: Thank you very much for these suggestions. We have re-conducted this assay and generated the new data in the revision (Figure 5B). The quantitative statistical analysis related to the ubiquitination levels of UBP14 was also conducted, and the results were included in Figure 5B in the new revision.

Minor:
1. Some sentences are not smooth, for example Line 144-145, Line 163, Line 207-208.

Response: Thank you for bringing this to our attention. We have re-organized the identified sentences and improved the writing of this manuscript through the use of a native English editing expert. Please see Line 143-144, Line 167-169, Line 209-211.

2. Please organize the pictures in an order according to the text. For example Line 187 Figure 3D, E.

Response: Thank you for your suggestions. We have examined all the pictures, and re-organized them in order according to the text (Figure 3).

22-Sep-2023
Prof. Dr. Siyi Guo
State Key Laboratory of Crop Stress Adaptation and Improvement, Henan University
Kaifeng, Henan
China

MSID: PP2023-RA-00938R1
MS TITLE: MAC3A and MAC3B ubiquitinate UBP14 to regulate organ size in Arabidopsis thaliana

Dear Dr. Siyi Guo:

I am pleased to inform you that your revised manuscript "MAC3A and MAC3B ubiquitinate UBP14 to regulate organ size in Arabidopsis thaliana" has been accepted for publication in Plant Physiology. Your manuscript will be checked for consistency with journal formatting and image standards by one of our Science Editors, who will contact you shortly. They may have a number of queries and suggestions for you. Please keep in mind that they work to increase the online visibility of your article and its attraction for readers from the widest of backgrounds. You will have the opportunity to review any revisions, and I hope you will work constructively with them to get the most out of your research. You will also have the opportunity to review all changes when you receive the author proofs for your article.

Once validated for publication, your article will be sent to a Science Editor for a final review of your manuscript and source files. After your final files are uploaded, your article will appear in the next available issue and the online Preview version of your article will be posted after your signed Copyright and Checklist forms and final source files are received.

Please be sure to include the following in your manuscript file:
Harvard style citations
List of all author contributions and funding information at the beginning of manuscript after the titles and author list.
List all supplemental materials associated with the article, with titles, at the end of the ms, before the references and acknowledgments section.
Complete Figure Legends for all figures appearing in the article listed before the Literature Cited.

Thank you and congratulations on your Plant Physiology paper!

Sincerely,
Dr. Haiyun Ren
Monitoring Editor, Plant Physiology

=====
FIRST AUTHOR PROFILES
=====

We recognize the hard work that goes into being First Author of a Plant Physiology paper by publishing a "First Author Feature" for each First Author alongside your paper.

If the first author(s) would like to submit information for a feature on Facebook and Twitter, fill out the form by clicking on the link below:

Plant Physiology First Author Feature Form: https://forms.office.com/r/ZBCGZfhm8F
If you cannot access the form through the link above, please contact Rachel at rbelsky@aspb.org.

To upload an image of yourself to accompany your profile, please put your photo in the correct journal folder at the link below. You must label your image: Author First Name Last Name Article Title.

First Author Profile Image Dropbox: https://www.dropbox.com/scl/fo/xz029p7qjr2jml2rp2kmq/h?dl=0&rlkey=6fkxcaqmxx35ap152b2osy1fz

=====
IMPORTANT REMINDER: PEER REVIEW REPORTS

=====

If you opted to publish a peer review report along with your article during the original submission process, it will be prepared by the editorial staff and publicly posted with your manuscript, inside the zip file that contains any other supplemental material. As a reminder, the peer review report is a public record of all comments from editors and reviewers, as well as your prior responses, as you received them in the decision letters for each draft of your manuscript. If you agreed to publish this report and have changed your mind, or are not sure if you selected this option, please contact the editorial office as soon as possible before signing the license agreement from our publisher.

-- Reviewer comments:
Reviewer #1 (Comments for the Author):

The authors have addressed my concerns in this revised verion, I have no further comments on this manuscript.

Reviewer #2 (Comments for the Author):

The authors have answered all my questions.
They provided new data and improved the manuscript according to suggestions from these two reviewers.
So, I don't have any more concerns.
